# Heritability and family-based GWAS analyses of the *N*-acyl ethanolamine and ceramide plasma lipidome

**DOI:** 10.1093/hmg/ddab002

**Published:** 2021-01-12

**Authors:** Kathryn A McGurk, Simon G Williams, Hui Guo, Hugh Watkins, Martin Farrall, Heather J Cordell, Anna Nicolaou, Bernard D Keavney

**Affiliations:** Division of Cardiovascular Sciences, Faculty of Biology, Medicine and Health, Manchester Academic Health Science Centre, University of Manchester, Manchester M13 9NT, UK; Laboratory for Lipidomics and Lipid Biology, Division of Pharmacy and Optometry, Faculty of Biology, Medicine and Health, Manchester Academic Health Science Centre, University of Manchester, Manchester M13 9PG, UK; Division of Cardiovascular Sciences, Faculty of Biology, Medicine and Health, Manchester Academic Health Science Centre, University of Manchester, Manchester M13 9NT, UK; Division of Population Health, Health Services Research & Primary Care, Faculty of Biology, Medicine and Health, Manchester Academic Health Science Centre, University of Manchester, Manchester M13 9PL, UK; Division of Cardiovascular Medicine, Radcliffe Department of Medicine, University of Oxford, Oxford OX3 9DU, UK; Wellcome Centre for Human Genetics, University of Oxford, Oxford OX3 7BN, UK; Division of Cardiovascular Medicine, Radcliffe Department of Medicine, University of Oxford, Oxford OX3 9DU, UK; Wellcome Centre for Human Genetics, University of Oxford, Oxford OX3 7BN, UK; Population Health Sciences Institute, Faculty of Medical Sciences, Newcastle University, Newcastle upon Tyne NE1 7RU, UK; Laboratory for Lipidomics and Lipid Biology, Division of Pharmacy and Optometry, Faculty of Biology, Medicine and Health, Manchester Academic Health Science Centre, University of Manchester, Manchester M13 9PG, UK; Division of Cardiovascular Sciences, Faculty of Biology, Medicine and Health, Manchester Academic Health Science Centre, University of Manchester, Manchester M13 9NT, UK; Manchester Heart Centre, Manchester University NHS Foundation Trust, Manchester M13 9WL, UK

## Abstract

Signalling lipids of the *N*-acyl ethanolamine (NAE) and ceramide (CER) classes have emerged as potential biomarkers of cardiovascular disease (CVD). We sought to establish the heritability of plasma NAEs (including the endocannabinoid anandamide) and CERs, to identify common DNA variants influencing the circulating concentrations of the heritable lipids, and assess causality of these lipids in CVD using 2-sample Mendelian randomization (2SMR). Nine NAEs and 16 CERs were analyzed in plasma samples from 999 members of 196 British Caucasian families, using targeted ultra-performance liquid chromatography with tandem mass spectrometry. All lipids were significantly heritable (*h*^2^ = 36–62%). A missense variant (rs324420) in the gene encoding the enzyme fatty acid amide hydrolase (*FAAH*), which degrades NAEs, associated at genome-wide association study (GWAS) significance (*P* < 5 × 10^−8^) with four NAEs (DHEA, PEA, LEA and VEA). For CERs, rs680379 in the *SPTLC3* gene, which encodes a subunit of the rate-limiting enzyme in CER biosynthesis, associated with a range of species (e.g. CER[N(24)S(19)]; *P* = 4.82 × 10^−27^). We observed three novel associations between SNPs at the *CD83*, *SGPP1* and *DEGS1* loci, and plasma CER traits (*P* < 5 × 10^−8^). 2SMR in the CARDIoGRAMplusC4D cohorts (60 801 cases; 123 504 controls) and in the DIAGRAM cohort (26 488 cases; 83 964 controls), using the genetic instruments from our family-based GWAS, did not reveal association between genetically determined differences in CER levels and CVD or diabetes. Two of the novel GWAS loci, *SGPP1* and *DEGS1*, suggested a casual association between CERs and a range of haematological phenotypes, through 2SMR in the UK Biobank, INTERVAL and UKBiLEVE cohorts (*n* = 110 000–350 000).

## Graphical Abstract

**Graphical Abstract:**
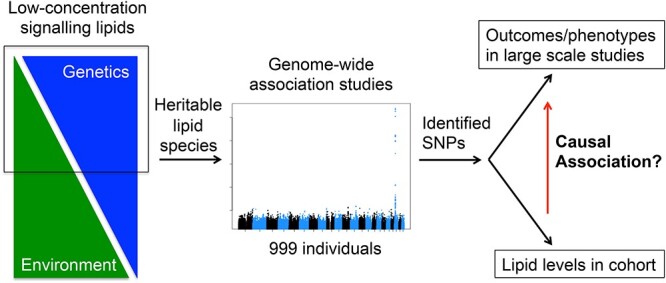

## Introduction

Genetic studies in large numbers of individuals have identified loci where common genetic variation influences the prevalence of plasma lipoproteins, such as high-density lipoprotein (HDL) and low-density lipoprotein (LDL), and plasma lipid species, such as triglycerides, cholesterol and polyunsaturated fatty acids (1–3). Recent advances in targeted lipidomics have enabled quantitative analyses of a greater proportion of the mediator lipidome in blood, supporting attempts to identify genetic associations for low-concentration bioactive lipid species to potentially find cardiovascular disease (CVD) biomarkers. Large-scale, untargeted metabolomic genome-wide association study (GWAS) have recently identified genetic variants of the major lipid-metabolising enzymes that influence the circulating levels of a few ceramide (CER) species, mainly derivatives of sphingosine, and the *N*-acyl ethanolamine (NAE) species oleoyl ethanolamide (OEA) (4–6).

**Figure 1:**
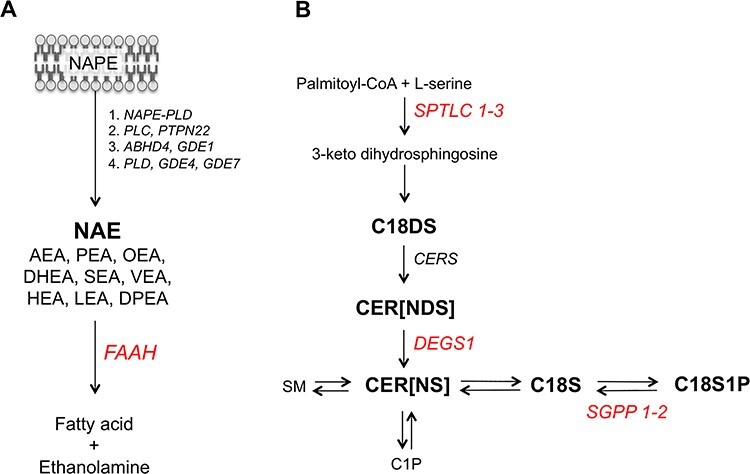
Schematic overview of the biosynthetic pathways for (**A**) *N*-acyl ethanolamines (NAEs) and (**B**) ceramides (CERs). (A) NAE species, including the endocannabinoid anandamide (AEA), are produced through four independent enzymatic pathways from the membrane phospholipid precursor (*N*-acyl phosphatidylethanolamine; NAPE). Fatty acid amide hydrolase (*FAAH*) degrades NAEs to free fatty acids (such as arachidonic acid for AEA) and ethanolamine. (B) CER species are biosynthesised via the enzyme serine palmitoyltransferase (*SPTLC 1-3*) that converts palmitoyl-CoA and L-serine to 3-keto dihydrosphingosine, in the rate-limiting step of the sphingolipid *de novo* pathway. The resulting dihydrosphingosine is coupled to various fatty acids via ceramide synthases (*CERS*) to generate dihydroceramides CER[NDS] that are further converted to CER[NS] via the enzyme delta 4-desaturase (*DEGS1*). Conversion of these pro-apoptotic CER[NS] species to sphingosine and sphingosine 1-phosphate, with roles in cell survival, degrades ceramides through reversible reactions. CER[NS] are also reversibly converted to sphingomyelin or further metabolized to ceramide 1-phosphate). Measured lipid species are in bold; genes encoding enzymes are in italics; genes identified through SNPs that associated at GWAS with circulating lipid levels are in red.

Bioactive lipids of the NAE and CER classes have potent roles in inflammation and immunity (7–9). NAEs are fatty acid derivatives, derived from membrane phospholipids and degraded by the enzyme fatty acid amide hydrolase (*FAAH*; Fig. 1). This class of bioactive lipids includes the endocannabinoid anandamide (AEA), the nuclear factor agonist palmitoyl ethanolamide (PEA) and a number of other species with intracellular roles in neuronal signalling and pain. Plasma levels of some NAEs are positively correlated with obesity (10–13).

CERs are members of the sphingolipid class, being derivatives of a sphingoid base (e.g. sphingosine and dihydrosphingosine) and fatty acids (Fig. 1). The first and also rate-limiting step of their *de novo* biosynthesis is catalyzed by the enzyme serine palmitoyltransferase, a heterodimeric protein whose monomers are encoded by the *SPTLC1-3* genes (14). CER plays important roles in apoptosis (15). Recently, some circulating CER species, derivatives of the 18-carbon base sphingosine (S18) and non-hydroxy fatty acids (e.g. CER[N(16)S(18)]) have been suggested as novel biomarkers of cardiovascular death (16), type-2 diabetes and insulin resistance (17). The contribution of genetic factors to plasma variation in some CER species has been investigated (5,6,18,19).

In this study we analyzed plasma NAEs and CERs by mass spectrometry-based targeted quantitative lipidomics in 196 British Caucasian families comprising 999 individuals, and determined their heritability and common genetic variant associations. We show that these bioactive lipid mediators are substantially heritable, and that plasma NAEs and a wide range of CERs are influenced by SNPs in key metabolic enzymes (*FAAH*, *SPTLC3*, *DEGS1* and *SGPP1)*. Furthermore, we identify a novel inflammatory locus (*CD83*) associated with CER species, and implicate this group of bioactive lipid mediators in haematological phenotypes through two-sample Mendelian randomization (2SMR), using SNPs in *DEGS1* and *SGPP1* as instruments. 2SMR in the CARDIOGRAMplusC4D cohorts (60 801 cases; 123 504 controls) and in the DIAGRAM cohort (26 488 cases; 83 964 controls), using the genetic instruments from our family-based GWAS, did not reveal association of NAE and CER species with CVD or diabetes.

## Results

### Heritability and GWAS associations of signalling lipid species

**Figure 2:**
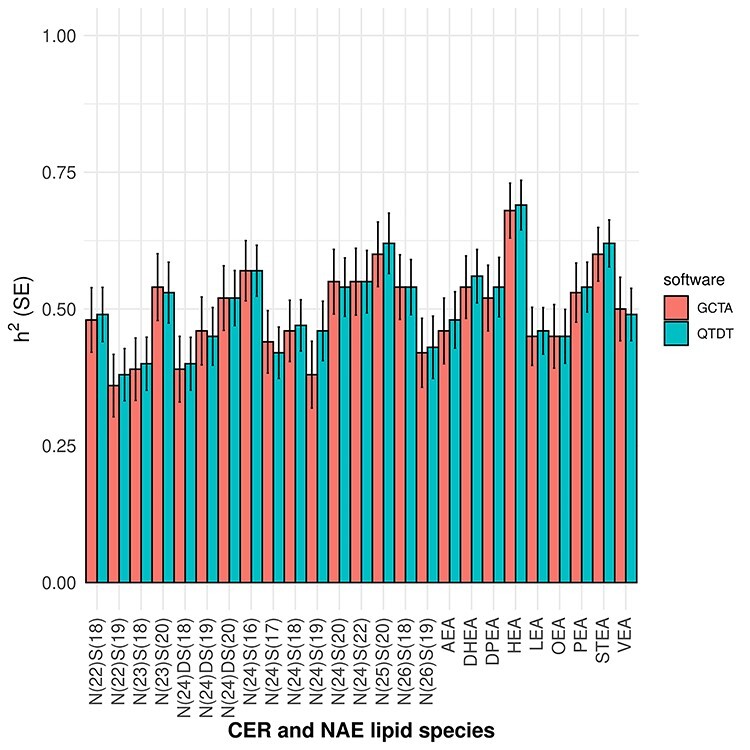
Heritability estimates of NAEs and CERs found in human plasma. This figure depicts the heritability estimated for each of 25 lipid species in 999 plasma samples using SNP-based GCTA software and reported pedigree-based QTDT software. These data are presented in detail in Supplementary Material, Table S8.

Plasma samples of 999 participants from 196 British Caucasian families were included in the genetic analyses. The families consisted of 1–24 members (mean of 5 members) with plasma available for lipidomics analyses (Supplementary Material, Fig. S2). Participant descriptions are listed in Table 1 and unadjusted concentrations of the lipids in Supplementary Material, Table S7. Of the nine NAE species identified in plasma, PEA was at highest abundance (1.89 ± 1.36 ng/ml [mean ± SD]), of the 16 plasma CER species, CER[N(24)S(18)] was most abundant (2.72 ± 1.29 nmol/ml), similar to healthy volunteers in previous studies (16,20–24). Heritability estimates of the signalling lipid species are presented Fig. 2 and Supplementary Material, Table S8. The NAE species had estimated heritabilities ranging from 45 to 69% (*P*_adj_ < 6.72 × 10^−15^), with *N-*heptadecanoyl ethanolamide (HEA) exhibiting the highest estimated heritability. The CER species had estimated heritabilities ranging from 36 to 62% (*P*_adj_ < 4.40 × 10^−13^), with CER[N(25)S(20)] exhibiting the highest estimated heritability.

**Table 1:** Summary statistics for the study participants

Trait	Mean (SD)
Gender	47% male
Hypertensive	33%
Mean blood pressure	138/83 mmHg
Age (years)	49 (15)
BMI	26.04 (4.33)
WHR	0.86 (0.09)
Cholesterol (mmol/L)	5.61 (1.20)

Data are shown as mean and standard deviation (SD) unless otherwise indicated; BMI, body mass index; WHR, waist-hip ratio.

**Figure 3:**
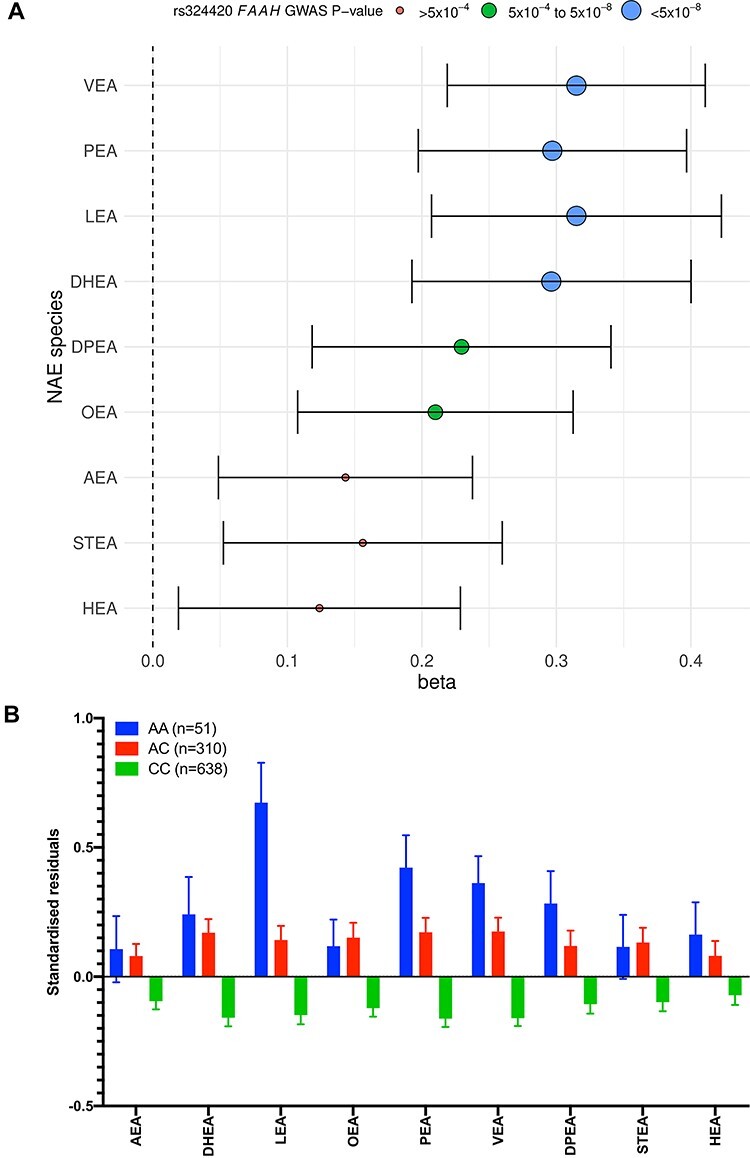

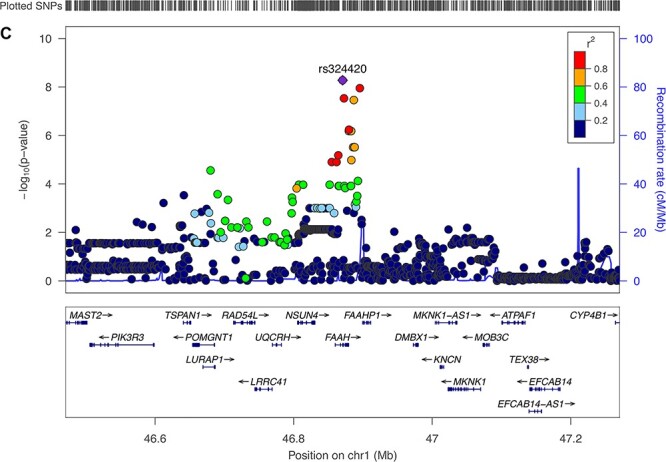
Family-based GWAS results for NAEs and the lead SNP in *FAAH*. (**A**) The forest plot depicts the effect (beta) and significance (GWAS *P*-value) for association between the lead SNP and eQTL of *FAAH* (rs324420) and each of 9 NAE species in 999 plasma samples. The *P*-values are grouped into ‘*P* < 5 × 10^−8^’, ‘*P* = 5 × 10^−4^–5.1 × 10^−8^’ and ‘*P* > 5 × 10^−4^’. (**B**) Trend in concentrations of plasma NAE species separated by FAAH rs324420 genotype. The figure depicts the mean standardized residuals of the NAE species in participants with the three genotypes at rs324420. The mean is shown with standard error. Fifty-one participants had the AA genotype, 310 had the AC genotype and 638 had the CC genotype in the cohort. (**C**) LocusZoom plot of the association of PEA with *FAAH* SNP rs324420. The LocusZoom plot depicts the association of NAE lipid species PEA with *FAAH* SNP rs324420 on chromosome 1 in 993 plasma samples. The *r*^2^ for each SNP is depicted in colour. The plot was created using the LocusZoom plot tools at http://locuszoom.sph.umich.edu/.

There were conventionally GWAS significant (*P* < 5 × 10^−8^) associations between four NAEs (*N*-docosahexaenoyl ethanolamide, DHEA; *N*-linoleoyl ethanolamide, LEA; *N*-palmitoyl ethanolamide PEA and *N*-vaccinoyl ethanolamide, VEA), as well as the sum of all NAEs (sumNAE), with SNPs in the gene encoding *FAAH*, which catalyzes the degradation of NAEs (Fig. 1, Supplementary Material, Table S9 and Supplementary Material, Fig. S3). The lead SNP is a missense variant (rs324420; C385A; P129T) and previously identified eQTL of *FAAH* in multiple tissues. Presence of the missense variant causes the enzyme to display normal catalytic properties but decreased cellular stability (25) by enhanced sensitivity of the enzyme to proteolytic degradation (20). The magnitude of the genetic effect was considerable (Fig. 3); the A allele of the lead SNP rs324420 caused a 0.23 SD per-allele increase in the plasma NAE species, and accounted for ~ 3% of the observed variability in associated NAEs. Unadjusted concentrations split by genotype status for the NAE species that associated to GWAS significance with SNPs in *FAAH* are presented in Supplementary Material, Table S14.

**Figure 4:**
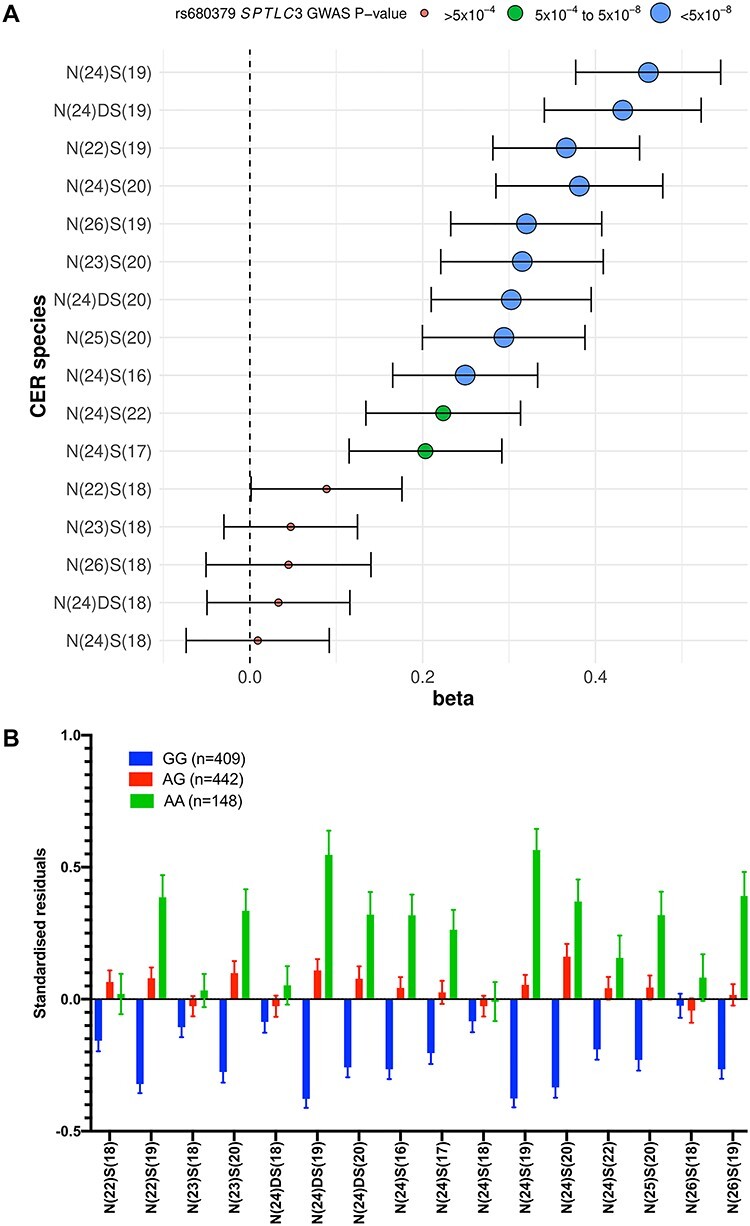

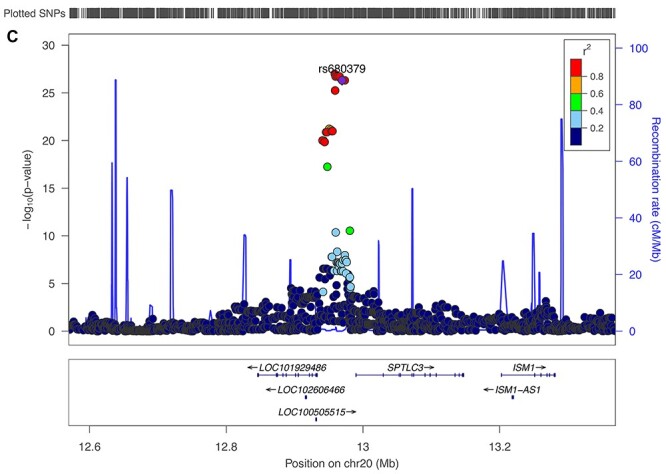
Family-based GWAS results for CER[NS] and precursor CER[NDS] with an exemplar SNP in serine palmitoyltransferase (*SPTLC3*). (**A**) The forest plot depicts the effect (beta) and significance (GWAS *P*-value) for association between the lead SNP and liver eQTL of *SPTLC3* (rs680379) with the 13 CER[NS] and 3 CER[NDS] species in 999 plasma samples. The *P*-values are grouped into ‘*P* < 5 × 10^−8^’, ‘*P* = 5 × 10^−4^–5.1 × 10^−8^’ and ‘*P* > 5 × 10^−4^’. (**B**) Trend in concentrations of plasma CER species separated by *SPTLC3* rs680379 genotype. The figure depicts the mean standardized residuals of the ceramide species in participants with the three genotypes at rs680379. The mean is shown with standard error. Four hundred nine participants had the GG genotype, 442 had the AG genotype and 148 had the AA genotype in the cohort. (**C**) LocusZoom plot of the association of CER[N(24)S(19)] with *SPTLC3* SNP rs680379. The LocusZoom plot depicts the association of CER[N(24)S(19)] with *SPTLC3* SNP rs680379 on chromosome 20 in 991 plasma samples. Although there is a group of lead SNPs, this SNP was depicted as it has been identified previously to associate at GWAS with sphingolipid species. The *r*^2^ for each SNP is depicted in colour. The plot was created using the LocusZoom plot tools at http://locuszoom.sph.umich.edu/.

Seven CER[NS] and two CER[NDS] species were significantly associated with SNPs in an intergenic region on chromosome 20 (Fig. 4, Supplementary Material, Fig. S4 and Supplementary Material, Table S10), lying 20 000 bases downstream of the gene encoding the third subunit of serine palmitoyltransferase (*SPTLC3*), which catalyzes the rate-limiting step (14) of CER biosynthesis (Fig. 1). Assessing the SNPs using GTEx confirmed them as the most significant liver eQTLs of *SPTLC3* (GTEx *P* = 5.7 × 10^−21^; NES = −0.47; Supplementary Material, Table S11). Associated SNPs had considerable phenotypic effects, for example the A allele of the SNP rs680379 was associated with a 0.30 SD per-allele increase in plasma CER, and was responsible for ~5% of the observed variability of associated CERs. Furthermore, the summed total of all CER species with 24-carbon non-hydroxy fatty acids, and, independently, those with 19- and 20-carbon sphingosine bases, were associated with the same SNPs at the *SPTLC3* locus (Supplementary Material, Table S10). Unadjusted concentrations split by genotype status for the CER species that associated to GWAS significance with SNPs in *SPTLC3* are presented in Supplementary Material, Table S13.

A novel association was identified for CER[N(26)S(19)] at a locus on chromosome 6, downstream of the gene for inflammatory protein CD83 (e.g. rs6940658, *P* = 2.07 × 10^−8^; Supplementary Material, Fig. S5 and Supplementary Material, Table S10)*.* A second novel association was found between SNPs upstream of the gene encoding sphingosine-1 phosphate phosphatase (*SGPP1*) and CER[N(24)S(16)] (e.g. rs7160525, *P* = 5.67 × 10^−10^; Supplementary Material, Fig. S7). This enzyme is involved in the recycling of CER[NS] species from sphingosine and sphingosine-1-phosphate (Fig. 1).

The product-precursor ratio of CER[NS] to CER[NDS] activity is indicative of delta 4-desaturase, sphingolipid 1 (*DEGS1*) activity (Fig. 1). To explore potential associations with *DEGS1* activity, two product-precursor ratios were available for analysis. First, the ratio of the product CER[N(24)S(19)] to its biochemical precursor CER[N(24)DS(19)], was associated with a set of SNPs in the upstream region of the *DEGS1* gene on chromosome 1 (e.g. rs4653568, beta = 0.29, *P* = 2.36 × 10^−10^, Supplementary Material, Fig. S6 and Table S10). All significant SNPs were confirmed eQTLs of *DEGS1* (Supplementary Material, Table S11). Individual GWAS of the precursor and product CER species did not associate with this locus to GWAS significance, however the association was stronger for the precursor CER[N(24)DS(19)] (*P* = 4.22 × 10^−7^, beta = −0.24) compared to the product CER[N(24)S(19)] (*P*-value = 0.07, beta = −0.08). Second, the ratio between the precursor lipid CER[N(24)DS(20)] and product CER[N(24)S(20)], did not yield a GWAS significant association, but tended to support the hypothesis that these SNPs are associated with *DEGS1* activity influencing the CER pathway (*P* = 2.76 × 10^−6^, beta = 0.21 for the ratio; *P* = 0.00046, beta = −0.16 for the precursor CER[NDS]; *P* = 0.48, beta = −0.03 for the product CER[NS]). Of note, and further supporting this hypothesis, a recent article identified a rare variant in *DEGS1* to influence plasma CERs (26).

### Two-sample Mendelian randomization analyses

We assessed the relationships between SNPs identified in our family cohort as significantly associated with NAE and CER levels, and both disease and metabolic phenotypes measured in the GeneAtlas Browser of PheWAS in the UK Biobank (27), the NHGRI-EBI GWAS catalogue (28) and PhenoScanner (29,30). This aimed to confirm that the proposed genetic instruments for 2SMR were specific. These analyses are presented in Supplementary Material, Table S9 for NAE-associated SNPs and Supplementary Material, Table S11(B) for CER-associated SNPs. Significant associations found were between all SNPs in *SGPP1* and *DEGS1* and a range of red cell and platelet phenotypes which included mean platelet volume, platelet count and reticulocyte percentage (*P* < 5 × 10^−8^, −0.03 > beta < 0.03; Supplementary Material, Table S11(B)). Of note, 4 of the 46 SNPs that associated with CER species in the loci of *SPTLC3* were associated with LDL cholesterol (*P* < 5 × 10^−8^, −0.03 > beta < 0.03; Supplementary Material, Table S11(B)).

2SMR analyses using the significant SNPs identified for *FAAH* (NAEs), *SPTLC3* (CERs)*, CD83* (CERs), *SGPP1* (CERs) and *DEGS1* (CERs), as genetic instruments, in the CARDIOGRAMplusC4D cohort of 60 801 coronary artery disease cases and 123 504 controls (31), did not indicate a causal role of the NAE and CER species for which we detected GWAS significant association, in CAD (*P* > 0.05 for all). Neither did we find significant evidence for a causal association of the NAE and CER species with type-2 diabetes using associated SNPs in these genes (*P* > 0.05 for all), in 2SMR analyses in the DIAGRAM cohort of 26 488 cases and 83 964 controls (32).

All SNPs significant for CER associations in our family cohort at the *SGPP1* locus and the *DEGS1* locus associated with a variety of blood cell phenotypes, identified in the UK Biobank GeneAtlas data (e.g. for CER[N(24)S(16)]: rs7160525 and mean platelet volume, *P* = 3.28 × 10^−29^; and for the product-precursor ratio at *DEGS1:* rs4653568 and mean platelet volume; *P* = 4.77 × 10^−12^; Supplementary Material, Table S11(B)). The statistically significant SNPs were used as instruments for single SNP 2SMR of blood cell phenotypes using published GWAS data sources (Supplementary Material, Table S6). The SNPs in *SGPP1* that associated with CER[N(24)S(16)] were significant (*P*_adj_ < 0.05) in influencing platelet and red blood cell traits. The SNPs in *DEGS1* that associated with the product/precursor ratio of CER[N(24)S(19)] to CER[N(24)DS(19)] was significant (*P*_adj_ < 0.05) in influencing mean platelet volume. Single SNPs remained associated to statistical significance when adjusted for multiple comparisons, however the effect sizes were small (−0.09 > beta <  0.09) (Supplementary Material, Table S12).

To address the question whether the principal genotype–phenotype associations for the CER SNPs could be with blood cell phenotypes, with the plasma associations being secondary, we generated a polygenic risk score (PRS) for mean platelet volume, one of the blood cell phenotypes most robustly associated with CER SNPs, and applied this to the plasma CER levels in the family cohort. The PRS was not significant (*P* > 0.05) in explaining the variation in CER[N(24)S(16)], nor the CER[N(24)S(19)]/CER[N(24DS(19)] product-precursor ratio. There was a weak correlation between the mean platelet volume PRS with either CER[N(24)S(16)] levels (Spearman’s rho = 0.03), or the product-precursor ratio (Spearman’s rho = 0.02), indicating that collectively, blood cell phenotype SNPs are at best very weakly associated with plasma CER levels and not supporting the hypothesis of a secondary association between genotypes and plasma levels due to a primary genotype-mediated effect on blood cells.

## Discussion

We show substantial heritability estimated for an array of signalling lipid mediators found in plasma and we identify GWAS significant associations between lipid species and DNA variants of the lipid metabolising enzymes in their respective metabolic pathways. We have provided the first GWAS significant evidence of association between SNPs in the *FAAH* gene and four plasma NAEs (DHEA, LEA, PEA and VEA). Additionally, we have extended the previously described association between SNPs in the *SPTLC3* gene and plasma CER to a wider range of species. Common variants in these two genes account for ~3% of the variation in associated NAEs and ~5% of the variation in associated CERs, respectively. In addition, we have shown novel SNP associations (*CD83*, *SGPP1* and *DEGS1)* influencing plasma CER species; two of these (*SGPP1* and *DEGS1*), potentially implicate these bioactive lipids in haematological phenotypes including mean platelet volume, red blood cell distribution width and reticulocyte fraction of red blood cells. 2SMR did not yield evidence for any causal effect of NAEs or CERs on coronary artery disease or type-2 diabetes.

The NAE species DHEA, LEA, PEA and VEA, associated with the SNP rs680379, a missense change in the NAE degradation enzyme FAAH. The association with PEA was identified previously in a single candidate gene study of mutations in *FAAH* in 114 subjects (33), which reported the same direction of effect on plasma AEA, PEA and OEA species but with *P*-values insignificant at genome-wide levels (0.003 < *P* < 0.04). OEA is the only NAE species that has been previously associated with DNA variants at GWAS significance. An eQTL of *FAAH* (rs1571138, upstream to *FAAHP1*, *P* = 5.15 × 10^−23^) that is in complete linkage disequilibrium (LD) with the lead SNP in our study, was identified in an untargeted lipidomics analysis of blood lipids; OEA was the only NAE species measured in that study (4)*.* Here, we found only a suggestive association with OEA (*P* = 5.80 × 10^−5^), although we observed non-significant trends in the same direction for all NAE species with genotype at this SNP (Fig. 3).

Although the *FAAH* missense SNP rs324420 is not associated with any disease endpoints identified from GWAS to date, the A allele, associated with higher NAE levels, has been reported to increase the risk of polysubstance addiction and abuse [MIM: 606581] in three candidate gene studies totalling 863 cases and 2170 controls (20,34,35). PheWAS analysis using the Gene Atlas UK Biobank online browser however, did not identify significant association in a similar number of UK Biobank cases of substance abuse/dependency (OR for A allele = 1.10; *P* = 0.14; 746 cases and 451 518 controls). The potential implication of NAE species in addiction through the association with the *FAAH* SNP, warrants further investigation in larger numbers of cases.

Narrow-sense heritability for CERs in previous studies has been estimated at between 9 and 51% (18,19). Here, we have assessed a more extensive array of species to show that further CER[NS] and CER[NDS] are significantly heritable. The novel association between the rs7157785 variant in sphingosine 1-phosphate phosphatase 1 (*SGPP1*), a CER metabolic enzyme, and CER[N(24)S(16)] is consistent with the enzyme’s role in influencing CER[NS] production, through the formation of sphingosine for CER[NS] biosynthesis (Fig. 1). All SNPs identified at this locus associated with haematological phenotypes in the UKBiobank PheWAS data. CERs have been previously shown to stimulate erythrocyte formation through platelet activating factor (36). However, further studies will be required to identify the mechanism of the association between genetically determined plasma CER levels and blood cell phenotypes.

CER[N(26)S(19)] associated at GWAS significance with SNPs at a novel locus on chromosome 6, downstream to the gene encoding the inflammatory protein CD83 (*P* = 2.07 × 10^−8^), a member of the immunoglobulin superfamily of membrane receptors expressed by antigen-presenting white blood cells, leukocytes and dendritic cells (37). An interaction between CD83 and CERs is currently unknown, but given the involvement of CER signalling in inflammation and immunity (7,38), it would be of interest to investigate further.

Association between some CER species and the *SPTLC3* SNP rs680379 has been identified previously through the use of shotgun lipidomics for four CER species (CER[N(22)S(18)], CER[N(23)S(18)], CER[N(24)S(18)] and CER[N(24:1)S(18)]) at GWAS significance (5,6). Here, we identify associations between an additional seven CER[NS] and two CER[NDS] plasma species and this SNP, and with other eQTLs of serine palmitoyltransferase at the same locus; as this enzyme is the rate-limiting step for the *de novo* CER biosynthesis, this association may have wider implications.

Although we did not find a significant association between all CER species and *SPTLC3* SNPs at GWAS significance, we observed non-significant trends in the same direction for all CER species with genotype at the rs680379 SNP (Fig. 4). The information gathered from the eQTL analysis highlights all of the *SPTLC3* confirmed eQTLs act in the liver, which is a major site for plasma CER biosynthesis. Neither PheWAS analysis in UK Biobank, nor 2SMR analysis, identified significant disease associations with the *SPTLC3* locus. A number of CER[NS] species have been studied as potential biomarkers of CVD and diabetes (16,39), and data from others have suggested that the *SPTLC3* locus is associated with these CERs (5,6). The extent to which specific species have a role in CVD remains debated (40,41).

Recent epidemiological studies have highlighted circulating CERs as novel biomarkers of coronary artery disease, type-2 diabetes and insulin resistance. There is a plausible case that CER[NS] may be implicated in atherosclerosis, as they are part of the lipid cargo of lipoprotein complexes, may aid the progression of plaques and lesions, and total CER levels are positively associated with total cholesterol and triglycerides (42–47). Although individual CER levels do not have strong evidence of association with disease, ratios of CERs containing a smaller fatty acid (such as CER[N(16)S(18)]) to CER[N(24)S(18)]) were increased in patients with fatal CAD outcome and in those experiencing acute coronary syndrome (16,41,48–51).

CERs have also been implicated in type-2 diabetes as novel biomarkers of insulin resistance. In an animal model, inhibition of serine palmitoyl transferase (Fig. 1) using the drug inhibitor myriocin, ameliorated glucocorticoid-, saturated fat- and obesity-induced insulin resistance (52). A number of clinical studies have reported associations between CER[NS] species and type-2 diabetes risk, incidence, and/or disease markers; such as fasting glucose and insulin, HOMA-IR and HOMA-B, and insulin sensitivity. However, the relationship between circulatory CERs and both CVD and type-2 diabetes, is not supported by all studies, and cannot as yet be tested in clinical trials as no CER specific drug is available, prompting our genetic investigation (17,39,40,53–56).

Notwithstanding the epidemiological associations summarized previously, we did not find evidence for a causal association between the plasma lipids analyzed in this project and coronary artery disease, myocardial infarction or diabetes through 2SMR. Four of the 46 SNPs that associated with plasma CERs at the *SPTLC3* locus had previously been identified in GWAS of LDL cholesterol. This suggests that there is a complex relationship between LDL cholesterol and CER species, which merits further investigation. Some SNPs (in *SGPP1* and *DEGS1*) were associated both with CER levels in the family cohort and with blood cell phenotypes involving red cells and platelets in metabolite GWAS data. Although 2SMR analyses were consistent with a causal effect of plasma CER levels on blood cell phenotypes, we cannot rule out a pleiotropic effect of these genes, through different mechanisms, on plasma lipid levels and blood cell phenotypes. SNPs in our most strongly associated genes accounted for ~3–5% of the plasma trait variability. Limited effect sizes of SNPs on an exposure can result in weak instrument bias when using smaller sample sizes, inflating the estimate towards the observational association or causing an association by chance (57). Further studies of the gene/lipid associations in larger numbers of participants will allow for stronger genetic instruments to be used as additional loci are discovered.

This study has certain limitations. First, although it is the largest study of which we are aware that has analyzed this range of plasma NAE and CER species, the number of samples studied (*n* = 999) limits the power to detect smaller genetic effects. Second, although samples were collected on ice and centrifuged generally within 4 h of collection, we observed some haemolysis in 14% of samples, which could have added random variability to the lipid measurements due to release of NAEs and CERs from damaged or lysed blood cells. Third, we did not have clinical information regarding lipid-lowering medication or hyperlipidaemia diagnoses. Fourth, non-fasting plasma samples were analyzed. Certain lipid mediators including NAEs can be altered with diet (58,59). These issues may have diminished our power to detect genetic associations, although some may not have major impact, for example, previous studies of blood CER using lipidomics analyses did not find differences between serum and plasma samples, and fasting and non-fasting samples (50,60,61).

In conclusion, this work discovered novel genetic associations with signalling lipids of the NAE and CER class but it did not provide confirmatory evidence for a causal role of NAEs or CERs in coronary artery disease or diabetes. Identification of additional loci affecting NAEs and CERs in larger lipidomics investigations, and in ethnically diverse populations, would be of great interest.

## Materials and Methods

### Family recruitment

Families were recruited for a quantitative genetic study of hypertension and other cardiovascular risk factors, and selected via a proband with essential hypertension (secondary hypertension was excluded using standard clinical criteria) as previously described (62). Probands were recruited from outpatients attending the John Radcliffe Hospital, Oxford hypertension clinic or via their family doctors. Included family members were UK residents of self-reported White European ancestry and were required to consist of ≥3 siblings quantitatively assessable for blood pressure if one parent of the sibship was available for blood sampling, or ≥4 siblings if no parent was available. The hypertensive proband could be either in the sibship or parental generation. First, second and third degree relatives were then recruited to assemble a series of extended families. The participants were fully phenotyped for blood pressure (using ambulatory monitoring), cardiovascular risk factors, blood biochemical measures and anthropometric traits. Non-fasting blood samples were collected into EDTA anticoagulant, kept on ice until centrifuged, and plasma was separated and stored at −80°C until lipidomic extraction. DNA was extracted from whole blood by standard methods. The collection protocol obtained ethical clearance from the Central Oxford Research Ethics Committee (06/Q1605/113) and it corresponds with the principles of the Declaration of Helsinki. Written informed consent was obtained from all participants. This cohort of extended families has previously been shown to have adequate power to detect moderate-sized genetic influences on quantitative traits (63–65).

### UPLC/ESI-MS/MS mediator lipidomics

Plasma samples were extracted and analyzed by mass spectrometry as previously described (59,66,68). Briefly, lipids were extracted from plasma (1 mL) using chloroform–methanol in the presence of internal standards: CER[N(25)S(18)] (50 pmol/sample; Ceramide/Sphingoid Internal Standard Mixture I, Avanti Polar Lipids, USA) for CER and AEA-*d*8 (20 ng/sample; Cayman Chemical Co., USA) for NAE. Targeted lipidomics was performed on a triple quadrupole mass spectrometer (Xevo TQS, Waters, UK) with an electrospray ionization probe coupled to a UPLC pump (Acquity UPLC, Waters, UK). CER species were separated on a C8 column (2.1 × 100 mm) and NAE were separated on a C18 column (2.1 × 50 mm) (both Acquity UPLC BEH, 1.7 μm, Waters, UK). NAE species were quantified using calibration lines of synthetic standards (Cayman Chemical); relative quantitation of CER was based on the use of internal standards (Avanti Polar Lipids) (68). Thus, the concentration of plasma NAE species are reported in pg/ml, whereas the relative abundance of plasma CER species is reported in pmol/ml. Pooled plasma samples from healthy volunteers were used to create quality control samples that were extracted and analyzed blindly alongside the familial samples. Detailed quality control information can be found in the Supplementary Material, Methods (Supplementary Material, Tables S1–S3). The CER[NS] notation (69) denotes a CER that contains a non-hydroxy fatty acid attached to a sphingosine base, for example, a 24-carbon non-hydroxy fatty acid joined to a 18-carbon sphingoid base is denoted as CER[N(24)S(18)], where N(24) represents a 24-carbon non-hydroxy fatty acid, and S(18) represents a 18-carbon sphingosine base attached. This species is also denoted as Cer(d18:1/24:0) in literature.

## Statistical analysis

### Covariate adjustment

Systematic error was considered from a variety of sources and assessed for collinearity; mass spectrometry batch and a trait created to adjust for sample issues (haemolysis or presence of white blood cells; present in 14% of samples) were included as potential covariates. Ascertainment selection was modelled via binary hypertension status. We found no substantial correlation with blood pressure traits and lipid levels (Supplementary Material, Table S4(B)). The resulting concentrations for each lipid species from the pooled quality control samples were used for adjustment of systematic errors during extraction, quantitation and data processing. The final set of potential covariates included mass spectrometry batch, sample abnormality, quality control sample measures, age, age^2^, sex, hypertension status, BMI and total cholesterol. The lipid measurements were assessed for effect of potential covariates using stepwise multiple linear regression to identify the best set of predictors, using the ‘caret’ package and ‘leapSeq’ method in R (version 3.5.2) (see Supplementary Material, Table S4 for predictors). Multiple linear regression of the best predictors was undertaken using the ‘lm’ function in R. Residuals from the covariate-adjusted regression models were standardized to have a mean of 0 and a variance of 1. Outliers were assessed using the R package ‘car’, assessing each observation by testing them as a mean-shift outlier based on studentized residuals, to remove the most extreme observations (Bonferroni *P*-value of *P* < 0.05). Missing values were coded as such in the genetics analyses. As lipid mediators can exert individual bioactivities, all lipid species were treated uniquely for all analyses, intra-class correlation analyses are depicted in Supplementary Material, Figure S1.

### Genome-wide genotyping quality control

Genotyping was performed using the Illumina 660W-Quad chip on 1234 individuals (580 males and 654 females) including 248 founders, at 557,124 SNPs. Quality control of the genotyping data was undertaken using PLINK (70) (version 1.9). No duplicate variants were found. SNPs that were identified as Mendelian inconsistencies (--Mendel-multigen) were marked as missing. Gender checks assessed by F-statistic (--check-sex) showed that gender as inferred from 538 771 chromosomal SNPs agreed with reported status. SNPs with low genotyping rates (--geno 0.05), low minor allele frequency (--maf 0.01) and those that failed checks of Hardy–Weinberg equilibrium (--hwe 1e-8) were excluded. Individuals with low genotype rates (--mind 0.05) and outlying heterozygosity were removed (0.31–0.33 included). Relatedness was assessed by high levels of IBD sharing (--genome and --rel-check) and by visualization of pairs of individuals’ degree of relatedness (through plotting the proportion of loci where the pair shares one allele IBD (Z1) by the proportion of loci where the pair shares zero alleles IBD (Z0)), and two outlier individuals were removed. Ethnicity was assessed via principal components analysis with genotype data from the 1000 Genome Project (71), which confirmed all participants were of European/CEU origin. Following quality control, 503 221 autosomal SNPs from 1219 individuals (216 families) were available for SNP-based heritability assessments, of which 999 individuals (196 families; 198 founders and 801 non-founders) had plasma available for lipidomics.

### Heritability estimates

SNP-based heritability was estimated using GCTA software (version 1.26.0) (72). A genetic relationship matrix was created from the quality controlled genotyping data and the --reml command was used to estimate variance of the traits explained by the genotyped SNPs. A complementary estimation of pedigree-based heritability was undertaken using the QTDT software (version 2.6.1) (73), by specifying the -we and -veg options to compare an environmental only variance model with a polygenic and environmental variances model. The *P*-values presented are adjusted for multiple comparisons via Bonferroni correction. Standard errors (SE) of the resulting narrow-sense heritability estimates from QTDT software were calculated by approximating the LRT as a Wald test. The least significant adjusted *P*-value from the groups of lipid species described are depicted as *P*_adj_ < X.

### Genotyping imputation

Following genotyping quality control, 503 221 autosomal SNPs were available to inform imputation. Imputation was performed through the Michigan Imputation Server (version v1.0.4), specifying pre-phasing with Eagle (74) (version 2.3) and imputation by Minimac3 (75) using the European population of the Human Reference Consortium (76) (version hrc.r1.1.2016). Following imputation, duplicate SNPs and SNPs with *r*^2^ < 0.8 were removed to generate a final set of 10 652 600 SNPs. Quality control was undertaken for the imputed data on the 999 individuals with lipidomics available, as follows: SNPs that were identified as Mendelian inconsistencies (--mendel-multigen) were marked as missing. SNPs with low call rates (--geno 0.05), low minor allele frequency (--maf 0.05) and those that failed checks of Hardy–Weinberg Equilibrium (--hwe 1e-8) were excluded, resulting in a final count of 5 280 459 SNPs available for genome-wide association analyses.

### Family-based genome-wide association studies

Linear mixed modelling approaches were used to account for family structure. Family-based genome-wide association analyses were undertaken for each lipid trait using GCTA software (version 1.26.0), specifying mixed linear model association analyses (--mlma). Genomic control inflation factors from the GWAS analyses can be found in Supplementary Material, Table S5. The least significant *P*-values of the significantly associated SNPs (*P* < 5 × 10^−8^) are depicted as *P* < X in the manuscript. Significantly associated SNPs were analyzed by Ensembl API Client (version 1.1.5 on GRCh37.p13) to identify neighbouring genes. Further analyses were undertaken of the significantly associated SNPs: variants were visualized using UCSC Genome Browser (77) and reviewed on OMIM (https://omim.org/); expression quantitative trait loci (eQTL) were identified using the GTEx eQTL Browser (version 8 (78)); and assessment of previously published GWAS and mGWAS associations with the detected SNPs was undertaken using the NHGRI-EBI GWAS Catalog, PhenoScanner and GeneAtlas UK Biobank PheWAS browser.

### Two-sample Mendelian randomization analysis

Two-sample Mendelian randomization (2SMR) analysis was undertaken in R following the guidelines provided by Smith *et al.* [https://mrcieu.github.io/TwoSampleMR/] (79). Selected examples of the significant associations identified for each class of lipid were analyzed by 2SMR for a number of previously published GWAS of interest. The GWAS significant associations (*P* < 5 × 10^−8^) identified were assessed for coronary artery disease (all), Type-2 Diabetes (all) and blood cell counts (CER[N(24)S(16)] and CER[N(24)S(19)]/CER[N(24)DS(19)] ratio). As many GWAS associated SNPs were in LD, clumping was undertaken using the 2SMR script [https://mrcieu.github.io/TwoSampleMR/articles/exposure.html]. SNPs were extracted from 1000 Genomes Project data, LD calculated between them and amongst those SNPs that have high R-sq, only the SNP with the lowest *P*-value was retained. The GWAS association beta of an exemplar lipid associated with each SNP was included in the analysis. The following SNPs remained after the data clumping step: rs324420 (*FAAH*; PEA); rs438568 (*SPTLC3*; CER[N(22)S(19)]); rs3848746 and rs7160525 (*SPTLC3* and *SGPP1*, respectively; CER[N(24)S(16)]); rs4653568 (*DEGS1*; CER[N(24)S(19)]/CER[N(24)DS(19)] ratio); and rs6940658 and rs438568 (*CD83* and *SPTLC3,* respectively; CER[N(26)S(19)].

2SMR was completed using available quantitative and disease outcome data from UK Biobank and multiple consortia such as CARDIOGRAMplusC4D, where the GWAS study with the maximum sample size was included in the analysis (Supplementary Material, Table S6). Due to the presence of only one or two independent GWAS significant SNPs per trait, 2SMR was assessed through Single SNP Mendelian Randomization (mr_singlesnp), of which Wald ratio is the method able to assess single SNP 2SMR. Outcomes included childhood obesity (80), type-2 diabetes (32), coronary heart disease (31), myocardial infarction (31), trunk fat mass, BMI, body fat percentage and blood cell traits (UK Biobank).

A PRS for mean platelet volume was created using the 423 SNPs reported on GWAS Catalog for association with this phenotype, from a GWAS of 746 667 individuals (81). In total, 329 variants were available following standard genotyping quality control of MAF > 0.01 and were found in the target dataset after correction for strand differences. PRS was generated and assessed as described by Choi *et al*. (2020), using PLINK [https://choishingwan.github.io/PRS-Tutorial/] (82). The PSR for mean platelet volume generated from publicly available data was tested for association with CER levels in our cohort.

## Supplementary Material

HMG-2020-EZ-00502_McGurk_Suppl_Clean_ddab002
