# Supplementary material for: Heritability and family-based GWAS analyses of the N-acyl ethanolamine and ceramide plasma lipidome
Source: Hum Mol Genet. 2021 Jan 12;30(6):500–13. doi: 10.1093/hmg/ddab002 (PMC8101358; doi:10.1093/hmg/ddab002)
Supplement: HMG-2020-EZ-00502_McGurk_Suppl_Clean_ddab002 [file hmg-2020-ez-00502_mcgurk_suppl_clean_ddab002.docx]

**Supplemental Materials**

**Supplemental methods**

**Quality assessment of plasma NAE species analysis**

Commercially available standards were used for peak referencing of the NAE species. Three pooled plasma samples (quality control; QC) were spiked with commercially available synthetic NAE standards added at a known concentration, and analysed in triplicate with blank ethanol injections run in between samples. Injection variation was analysed by calculation of the mean response and standard deviation of the triplicate injections for each of the three QC samples. The coefficient of variation was calculated, and the mean values are shown in Tables S1. A pooled sample was analysed alongside each batch of clinical samples and a species-specific response value was added to the adjustment model to account for inter-batch variability. An estimation of sample carry-over was calculated by assessing the presence of lipid species in blank injections; there was detectable carryover of NAE species.

**Table S1:** Mean injection coefficient of variation (CV) (n=3 samples) of plasma N-acyl ethanolamine (NAE) species.

| NAE | Mean  Injection  CV (%) |
| --- | --- |
| AEA | 4 |
| PEA | 4 |
| OEA | 6 |
| VEA | 6 |
| LEA | 3 |
| DPEA | 2 |
| DHEA | 6 |
| HEA | 4 |
| STEA | 15 |

**Quality assessment of plasma CER species analysis**

Due to lack of commercially available synthetic standards for every CER species detected in human plasma, CER were analysed via multiple reaction monitoring (MRM) using three transitions per target compound (Table S2). A pooled lipid extract sample was used for the identification of CER species, and the coefficient of variation was calculated (Table S3).

**Table S2:** Multiple reaction monitoring transitions used for plasma ceramide analysis. R = fatty acyl chain

| **Ceramide** | ***m/z*** | ***m/z*** | ***m/z*** | ***m/z*** | ***m/z*** |
| --- | --- | --- | --- | --- | --- |
|  | **Precursor** | **Fragment 1** | **Fragment 2** | **Fragment 3** | **Fragment 4** |
|  | **[M+H]^+^** | **[M+H-R-HCHO]^+^** | **[M+H-R-2H_2_O]^+^** | **[M+H-R-H_2_O]^+^** | **[M+H-R]+** |
| CER[N(22)S(18)] | 662.6 | 252.4 | 264.4 | 282.4 |  |
| CER[N(22)S(19)] | 636.6 | 266.4 | 278.4 | 296.4 |  |
| CER[N(23)S(18)] | 636.6 | 252.4 | 264.4 | 282.4 |  |
| CER[N(23)S(20)] | 664.7 | 280.4 | 292.4 | 310.4 |  |
| CER[N(24)DS(18)] | 652.7 |  | 266.4 | 284.4 | 302.4 |
| CER[N(24)DS(19)] | 666.7 |  | 280.4 | 289.4 | 316.4 |
| CER[N(24)DS(20)] | 680.7 |  | 294.4 | 312.4 | 330.4 |
| CER[N(24)S(16)] | 622.6 | 224.4 | 236.4 | 254.4 |  |
| CER[N(24)S(17)] | 636.6 | 238.4 | 250.4 | 268.4 |  |
| CER[N(24)S(18)] | 650.7 | 252.4 | 264.4 | 282.4 |  |
| CER[N(24)S(19)] | 664.7 | 266.4 | 278.4 | 296.4 |  |
| CER[N(24)S(20)] | 678.7 | 280.4 | 292.4 | 310.4 |  |
| CER[N(24)S(22)] | 706.7 | 308.4 | 320.4 | 338.4 |  |
| CER[N(25)S(20)] | 692.7 | 280.4 | 292.4 | 310.4 |  |
| CER[N(26)S(18)] | 678.7 | 252.4 | 264.4 | 282.4 |  |
| CER[N(26)S(19)] | 692.7 | 266.4 | 278.4 | 296.4 |  |

**Table S3:** Mean injection coefficient of variation (CV) (n=3 samples) of plasma ceramide species.

| Ceramide | Mean injection  CV (%) |
| --- | --- |
| CER[N(22)S(18)] | 3 |
| CER[N(22)S(19)] | 11 |
| CER[N(23)S(18)] | 5 |
| CER[N(23)S(20)] | 3 |
| CER[N(24)DS(18)] | 2 |
| CER[N(24)DS(19)] | 6 |
| CER[N(24)DS(20)] | 7 |
| CER[N(24)S(16)] | 5 |
| CER[N(24)S(17)] | 5 |
| CER[N(24)S(18)] | 6 |
| CER[N(24)S(19)] | 7 |
| CER[N(24)S(20)] | 6 |
| CER[N(24)S(22)] | 9 |
| CER[N(25)S(20)] | 7 |
| CER[N(26)S(18)] | 3 |
| CER[N(26)S(19)] | 4 |

**Supplemental Tables**

**Table S4.** Predictors identified from stepwise-multiple linear regression.

A) qc, quality control sample; Batch, mass spectrometry batch; Age, age at enrolment; Abnormality, presence of white blood cells, red blood cells or other sample abnormality. B) Correlation coefficient (R) of association between CER levels and hypertension status and blood pressure traits. We found no substantial correlation with blood pressure traits and lipid levels (-0.15<R<0.15). C) As anticipated, the significance of genetic effects on the lipids for which cholesterol and BMI were significant covariates, were somewhat diminished in analyses unadjusted for the two covariates. The table depicts the association of the lipids with the lead SNPs in *FAAH* and *SPTLC3* without the inclusion of cholesterol or BMI.

**A)**

| **Class** | **Lipid** | **Predictors** |
| --- | --- | --- |
| CER | N(22)S(18) | N22_S18_qc + Batch + Abnormality + Age + Sex + Hypertension + Age2 |
| CER | N(22)S(19) | N22_S19_qc + Batch + Abnormality + Cholesterol |
| CER | N(23)S(18) | N23_S18_qc + Batch + Age2 + Age |
| CER | N(23)S(20) | N23_S20_qc + Batch + Sex + Age2 |
| CER | N(24)DS(18) | N24_DS18_qc + Batch + Sex + Age + BMI + Cholesterol |
| CER | N(24)DS(19) | N24_DS19_qc + Batch + Sex + BMI + Cholesterol |
| CER | N(24)DS(20) | N24_DS20_qc + Batch + Abnormality + Sex + Hypertension + Age + Age2 + BMI + Cholesterol |
| CER | N(24)S(16) | N24_S16_qc + Batch + Abnormality + Sex + Age + Age2 |
| CER | N(24)S(17) | N24_S17_qc + Batch + Sex + Age + Age2 |
| CER | N(24)S(18) | N24_S18_qc + Batch + Age + Age2 + Cholesterol |
| CER | N(24)S(19) | N24_S19_qc + Batch + Abnormality |
| CER | N(24)S(20) | N24_S20_qc + Batch + Abnormality + Sex + Hypertension + Age + Age2 + BMI + Cholesterol |
| CER | N(24)S(22) | N24_S22_qc + Sex + Age2 + BMI |
| CER | N(25)S(20) | N25_S20_qc + Age + BMI + Cholesterol |
| CER | N(26)S(18) | N26_S18_qc + Batch + Sex + Age + Age2 + Cholesterol |
| CER | N(26)S(19) | N26_S19_qc + Sex + Age2 + BMI + Cholesterol |
| NAE | AEA | AEA_qc + Cholesterol + BMI + Batch |
| NAE | DHEA | DHEA_qc + Cholesterol + Age2 + Batch |
| NAE | DPEA | DPEA_qc + Cholesterol + BMI + Batch + Sex + Hypertension + Age2 + Age + Abnormality |
| NAE | OEA | OEA_qc + Cholesterol + Batch + BMI |
| NAE | LEA | LEA_qc + Cholesterol + Batch + Age2 |
| NAE | PEA | PEA_qc + Cholesterol |
| NAE | VEA | VEA_qc + Cholesterol + Batch + Sex + Hypertension |
| NAE | STEA | STEA_qc + Cholesterol + Batch + Sex + BMI |
| NAE | HEA | HEA_qc + Cholesterol + Batch |

**B)**

| Lipid | Hypertension  Status | Clinical Systolic  Blood Pressure | Clinical Diastolic  Blood Pressure |
| --- | --- | --- | --- |
| N(22)S(18) | -0.03 | -0.03 | -0.02 |
| N(22)S(19) | -0.03 | -0.02 | -0.03 |
| N(23)S(18) | -0.07 | -0.06 | -0.06 |
| N(23)S(20) | 0.01 | 0.01 | 0.01 |
| N(24)DS(18) | 0 | 0.03 | 0.07 |
| N(24)DS(19) | 0.04 | 0.05 | 0.12 |
| N(24)DS(20) | 0.03 | 0.03 | 0.11 |
| N(24)S(16) | 0.01 | 0.01 | 0.03 |
| N(24)S(17) | -0.1 | -0.08 | -0.06 |
| N(24)S(18) | -0.09 | -0.09 | -0.07 |
| N(24)S(19) | -0.08 | -0.05 | -0.04 |
| N(24)S(20) | 0.01 | 0.02 | 0.08 |
| N(24)S(22) | -0.07 | -0.1 | -0.05 |
| N(25)S(20) | 0.08 | 0.05 | 0.08 |
| N(26)S(18) | 0.06 | 0.05 | 0.08 |
| N(26)S(19) | 0.01 | 0.01 | 0.02 |
| AEA | -0.06 | 0.03 | 0.01 |
| DHEA | -0.01 | 0.06 | 0.02 |
| DPEA | -0.07 | 0.03 | 0 |
| HEA | -0.02 | 0.03 | 0.01 |
| LEA | -0.11 | -0.06 | -0.05 |
| OEA | -0.11 | -0.04 | -0.05 |
| PEA | -0.09 | 0 | -0.01 |
| STEA | -0.05 | 0 | -0.02 |
| VEA | -0.09 | -0.02 | -0.01 |

**C)**

| **Lipid** | **Beta** | **SE** | **P-value** | **SNP** |
| --- | --- | --- | --- | --- |
| DHEA | 0.28 | 0.05 | 1.74E-07 | rs324420 |
| LEA | 0.31 | 0.05 | 1.99E-08 | rs324420 |
| PEA | 0.28 | 0.05 | 2.97E-08 | rs324420 |
| VEA | 0.31 | 0.05 | 2.71E-10 | rs324420 |
| N(22)S(19) | 0.36 | 0.04 | 6.81E-17 | rs680379 |
| N(24)DS(19) | 0.42 | 0.05 | 8.08E-20 | rs680379 |
| N(24)DS(20) | 0.3 | 0.05 | 2.31E-10 | rs680379 |
| N(24)S(20) | 0.37 | 0.05 | 3.16E-14 | rs680379 |
| N(25)S(20) | 0.3 | 0.05 | 5.96E-10 | rs680379 |
| N(26)S(19) | 0.32 | 0.04 | 4.67E-13 | rs680379 |

**Table S5.** Genomic inflation factors (GIF) from GWAS results per trait.

| **Class** | **Lipid** | **GIF** |
| --- | --- | --- |
| CER | N(22)S(18) | 1.0042 |
| CER | N(22)S(19) | 0.9893 |
| CER | N(23)S(18) | 1.0000 |
| CER | N(23)S(20) | 1.0046 |
| CER | N(24)DS(18) | 1.0044 |
| CER | N(24)DS(19) | 0.9952 |
| CER | N(24)DS(20) | 1.0045 |
| CER | N(24)S(16) | 1.0012 |
| CER | N(24)S(17) | 1.0037 |
| CER | N(24)S(18) | 1.0036 |
| CER | N(24)S(19) | 0.9944 |
| CER | N(24)S(20) | 0.9907 |
| CER | N(24)S(22) | 1.0036 |
| CER | N(25)S(20) | 1.0061 |
| CER | N(26)S(18) | 1.0017 |
| CER | N(26)S(19) | 0.9939 |
| CER | n24s19ratio | 0.9937 |
| CER | s19_sum | 0.9954 |
| CER | s20_sum | 0.9945 |
| NAE | AEA | 0.9984 |
| NAE | DHEA | 0.9916 |
| NAE | DPEA | 1.0062 |
| NAE | LEA | 0.9910 |
| NAE | OEA | 0.9916 |
| NAE | PEA | 0.9904 |
| NAE | VEA | 0.9921 |
| NAE | STEA | 0.9984 |
| NAE | HEA | 0.9953 |
| NAE | sumEA | 0.9961 |

**Table S6.** List of GWAS assessed by 2SMR analysis for relationship with the SNPs identified at GWAS to associate with the lipid species.

id, 2SMR software ID; pmid, PubMed ID; pop, population type; EU, European.

| **author** | **consortium** | **id** | **ncase** | **ncontrol** | **nsnp** | **pmid** | **pop** | **sample**  **size** | **sex** | **trait** | **unit** | **year** |
| --- | --- | --- | --- | --- | --- | --- | --- | --- | --- | --- | --- | --- |
| Nikpay | CARDIoGRAM  plusC4D | 7 | 60801 | 123504 | 9455779 | 26343387 | Mix | 184305 | both | Coronary heart  Disease and MI | log  odds | 2015 |
| Mahajan | DIAGRAM | 23 | 26488 | 83964 | 2915012 | 24509480 | Mix | 110452 | both | Type 2  diabetes | Log  odds | 2014 |
| Astle | UK Biobank INTERVAL  UK BiLEVE | ebi-a-GCST004612 | NA | NA | 29163693 | 27863252 | EU | 170763 | both | Blood cell traits | NA | 2016 |
| Neale lab | UK Biobank | ukb-d-30100_irnt  ukb-d-30080_irnt  ukb-d-30290_irnt  ukb-d-30300_irnt  ukb-d-30280_irnt  ukb-d-30240_irnt | NA | NA | 13586285 | NA | EU | 350470 | both | Blood cell traits | NA | 2018 |
| Elsworth | UK Biobank | ukb-b-20044 | NA | NA | 9851867 | NA | EU | 454588 | both | Trunk fat  mass | SD | 2018 |
| Elsworth | UK Biobank | ukb-b-8909 | NA | NA | 9851867 | NA | EU | 454588 | both | Body fat  percentage | SD | 2018 |
| Bradfield | - | ieu-a-1096 | 5530 | 8318 | 2442739 | 22484627 | EU | 13848 | both | Childhood  obesity | NA | 2012 |
| Elsworth | UK Biobank | ukb-b-19953 | NA | NA | 9851867 | NA | EU | 454588 | both | Body mass  index (BMI) | SD | 2018 |
| Pilling | UK Biobank | ebi-a-GCST006804 | NA | NA | 16671830 | 28957414 | EU | 116666 | NA | Blood cell traits | NA | 2017 |

**Table S7:** Lipid species, corresponding class, summary statistics, and description of the species.

The concentrations are provided before adjustment or outlier removal for a total of 999 samples analysed from 196 families.

| **Lipid** | **Class** | **unit** | **N** | **Mean** | **SD** | **Description of lipid species** |
| --- | --- | --- | --- | --- | --- | --- |
| N(22)S(18) | CER | pmol/ml | 999 | 128 | 74 | Non-hydroxy fatty acid and sphingosine bas |
| N(22)S(19) | CER | pmol/ml | 999 | 32 | 22 | Non-hydroxy fatty acid and sphingosine base |
| N(23)S(18) | CER | pmol/ml | 999 | 866 | 365 | Non-hydroxy fatty acid and sphingosine base |
| N(23)S(20) | CER | pmol/ml | 999 | 51 | 16 | Non-hydroxy fatty acid and sphingosine base |
| N(24)DS(18) | CER | pmol/ml | 999 | 175 | 112 | Non-hydroxy fatty acid and dihydrosphingosine base |
| N(24)DS(19) | CER | pmol/ml | 999 | 66 | 42 | Non-hydroxy fatty acid and dihydrosphingosine base |
| N(24)DS(20) | CER | pmol/ml | 998 | 40 | 22 | Non-hydroxy fatty acid and dihydrosphingosine base |
| N(24)S(16) | CER | pmol/ml | 999 | 49 | 30 | Non-hydroxy fatty acid and sphingosine base |
| N(24)S(17) | CER | pmol/ml | 999 | 236 | 106 | Non-hydroxy fatty acid and sphingosine base |
| N(24)S(18) | CER | pmol/ml | 999 | 2724 | 1290 | Non-hydroxy fatty acid and sphingosine base |
| N(24)S(19) | CER | pmol/ml | 999 | 1068 | 486 | Non-hydroxy fatty acid and sphingosine base |
| N(24)S(20) | CER | pmol/ml | 999 | 249 | 93 | Non-hydroxy fatty acid and sphingosine base |
| N(24)S(22) | CER | pmol/ml | 999 | 45 | 31 | Non-hydroxy fatty acid and sphingosine base |
| N(25)S(20) | CER | pmol/ml | 999 | 38 | 19 | Non-hydroxy fatty acid and sphingosine base |
| N(26)S(18) | CER | pmol/ml | 999 | 706 | 219 | Non-hydroxy fatty acid and sphingosine base |
| N(26)S(19) | CER | pmol/ml | 999 | 108 | 74 | Non-hydroxy fatty acid and sphingosine base |
| AEA | NAE | pg/ml | 998 | 352 | 334 | Anandamide (N-arachidonoyl ethanolamide) |
| DHEA | NAE | pg/ml | 996 | 349 | 290 | N-docosahexaenoyl ethanolamide |
| DPEA | NAE | pg/ml | 990 | 22 | 17 | N-docosapentaenoyl ethanolamine |
| LEA | NAE | pg/ml | 999 | 619 | 511 | N-linoleoyl ethanolamide |
| OEA | NAE | pg/ml | 999 | 568 | 531 | N-oleoyl ethanolamide |
| PEA | NAE | pg/ml | 999 | 1884 | 1356 | N-palmitoyl ethanolamide |
| VEA | NAE | pg/ml | 999 | 252 | 259 | N-vaccinoyl ethanolamide |
| HEA | NAE | pg/ml | 968 | 24 | 19 | N-heptadecanoyl ethanolamide |
| STEA | NAE | pg/ml | 999 | 495 | 447 | N-stearoyl ethanolamide |

**Table S8.** Heritability of plasma lipid species measured.

Heritability was estimated using GCTA SNP-based software using the genotyping data and QTDT pedigree-based software. h^2^, estimated heritability; SE, standard error; n, number of individuals included in analysis after outlier removal; P-adj, P-value after Bonferroni adjustment of the P-value for multiple testing (16 for CER measured, 9 for NAE measures). A GCTA P-value of 0 is represented as a P-value result of <6.11x10^-16^.

|  |  | **QTDT** | | | | | **GCTA** | | | | |
| --- | --- | --- | --- | --- | --- | --- | --- | --- | --- | --- | --- |
| **Class** | **Lipid** | **h^2^** | **SE** | **n** | **P-value** | **P-adj** | **h^2^** | **SE** | **n** | **P-value** | **P-adj** |
| CER | N(22)S(18) | 0.49 | 0.049 | 993 | 5.00E-23 | 1.50E-21 | 0.48 | 0.059 | 993 | 6.11E-16 | 1.83E-14 |
| CER | N(22)S(19) | 0.38 | 0.048 | 992 | 1.00E-15 | 1.10E-14 | 0.36 | 0.057 | 992 | 6.11E-15 | 6.72E-14 |
| CER | N(23)S(18) | 0.40 | 0.049 | 992 | 2.00E-16 | 6.00E-15 | 0.39 | 0.057 | 992 | 1.11E-16 | 3.33E-15 |
| CER | N(23)S(20) | 0.53 | 0.056 | 994 | 1.00E-21 | 1.10E-20 | 0.54 | 0.061 | 994 | 6.11E-16 | 6.72E-15 |
| CER | N(24)DS(18) | 0.40 | 0.048 | 992 | 8.00E-17 | 2.40E-15 | 0.39 | 0.06 | 992 | 5.55E-17 | 1.67E-15 |
| CER | N(24)DS(19) | 0.45 | 0.053 | 994 | 1.00E-17 | 1.10E-16 | 0.46 | 0.062 | 994 | 6.11E-16 | 6.72E-15 |
| CER | N(24)DS(20) | 0.52 | 0.050 | 993 | 6.00E-25 | 6.60E-24 | 0.52 | 0.059 | 993 | 6.11E-16 | 6.72E-15 |
| CER | N(24)S(16) | 0.57 | 0.047 | 992 | 2.00E-34 | 2.20E-33 | 0.57 | 0.055 | 992 | 6.11E-16 | 6.72E-15 |
| CER | N(24)S(17) | 0.42 | 0.047 | 994 | 3.00E-19 | 3.30E-18 | 0.44 | 0.057 | 994 | 6.11E-16 | 6.72E-15 |
| CER | N(24)S(18) | 0.47 | 0.047 | 991 | 1.00E-23 | 1.10E-22 | 0.46 | 0.056 | 991 | 6.11E-16 | 6.72E-15 |
| CER | N(24)S(19) | 0.46 | 0.054 | 991 | 2.00E-17 | 2.20E-16 | 0.38 | 0.061 | 993 | 9.94E-15 | 1.09E-13 |
| CER | N(24)S(20) | 0.54 | 0.053 | 996 | 5.00E-24 | 5.50E-23 | 0.55 | 0.059 | 996 | 6.11E-16 | 6.72E-15 |
| CER | N(24)S(22) | 0.55 | 0.057 | 992 | 6.00E-22 | 6.60E-21 | 0.55 | 0.061 | 992 | 6.11E-16 | 6.72E-15 |
| CER | N(25)S(20) | 0.62 | 0.055 | 994 | 3.00E-29 | 3.30E-28 | 0.60 | 0.059 | 994 | 6.11E-16 | 6.72E-15 |
| CER | N(26)S(18) | 0.54 | 0.050 | 995 | 7.00E-27 | 2.10E-25 | 0.54 | 0.059 | 995 | 6.11E-16 | 1.83E-14 |
| CER | N(26)S(19) | 0.43 | 0.057 | 991 | 4.00E-14 | 4.40E-13 | 0.42 | 0.063 | 991 | 3.24E-14 | 3.56E-13 |
| NAE | AEA | 0.48 | 0.051 | 994 | 1.00E-20 | 7.00E-20 | 0.46 | 0.06 | 994 | 6.11E-16 | 4.28E-15 |
| NAE | DHEA | 0.56 | 0.049 | 990 | 2.00E-30 | 1.40E-29 | 0.54 | 0.057 | 990 | 6.11E-16 | 4.28E-15 |
| NAE | DPEA | 0.54 | 0.054 | 986 | 2.00E-23 | 1.40E-22 | 0.52 | 0.06 | 986 | 6.11E-16 | 4.28E-15 |
| NAE | LEA | 0.46 | 0.043 | 994 | 3.00E-27 | 2.10E-26 | 0.45 | 0.053 | 994 | 6.11E-16 | 4.28E-15 |
| NAE | OEA | 0.45 | 0.049 | 994 | 4.00E-20 | 2.80E-19 | 0.45 | 0.058 | 994 | 6.11E-16 | 4.28E-15 |
| NAE | PEA | 0.54 | 0.046 | 993 | 2.00E-32 | 1.40E-31 | 0.53 | 0.054 | 993 | 6.11E-16 | 4.28E-15 |
| NAE | VEA | 0.49 | 0.048 | 994 | 1.00E-24 | 7.00E-24 | 0.50 | 0.058 | 994 | 6.11E-16 | 4.28E-15 |
| NAE | STEA | 0.62 | 0.043 | 994 | 2.00E-47 | 2.20E-46 | 0.60 | 0.049 | 994 | 6.11E-16 | 6.72E-15 |
| NAE | HEA | 0.69 | 0.045 | 964 | 2.00E-52 | 2.20E-51 | 0.68 | 0.05 | 964 | 6.11E-16 | 6.72E-15 |

**Table S9.** Significant GWAS associations for *N*-acyl ethanolamine species.

A) Description of the GWAS significant associations identified. B) Description of the SNPs using the Ensembl API Client. C) Summary of the information on eQTL status as identified using the GTEX browser, including a section specifying whole blood only, GWAS Catalog and PhenoScanner previous associations, and Gene Atlas PheWAS.

| **A** |  |  |  |  |  |  |  |  |  |
| --- | --- | --- | --- | --- | --- | --- | --- | --- | --- |
| **Lipid** | **Chr** | **SNP** | **Position** | **A1** | **A2** | **MAF** | **Beta** | **SE** | **P-value** |
| DHEA | 1 | rs324420 | 46870761 | A | C | 0.20 | 0.30 | 0.053 | 2.15E-08 |
| DHEA | 1 | rs324422 | 46886782 | T | C | 0.23 | 0.28 | 0.050 | 2.76E-08 |
| DHEA | 1 | rs324418 | 46872698 | G | A | 0.22 | 0.28 | 0.052 | 4.36E-08 |
| LEA | 1 | rs324420 | 46870761 | A | C | 0.20 | 0.31 | 0.055 | 1.01E-08 |
| LEA | 1 | rs1571138 | 46895641 | A | G | 0.20 | 0.31 | 0.055 | 2.24E-08 |
| LEA | 1 | rs324422 | 46886782 | T | C | 0.23 | 0.29 | 0.052 | 2.32E-08 |
| PEA | 1 | rs324420 | 46870761 | A | C | 0.21 | 0.30 | 0.051 | 5.30E-09 |
| PEA | 1 | rs1571138 | 46895641 | A | G | 0.20 | 0.29 | 0.051 | 1.13E-08 |
| PEA | 1 | rs324418 | 46872698 | G | A | 0.22 | 0.27 | 0.050 | 2.96E-08 |
| PEA | 1 | rs324422 | 46886782 | T | C | 0.23 | 0.27 | 0.048 | 3.44E-08 |
| VEA | 1 | rs324420 | 46870761 | A | C | 0.20 | 0.31 | 0.049 | 1.24E-10 |
| VEA | 1 | rs1571138 | 46895641 | A | G | 0.20 | 0.32 | 0.049 | 1.25E-10 |
| VEA | 1 | rs324418 | 46872698 | G | A | 0.22 | 0.29 | 0.048 | 1.15E-09 |
| VEA | 1 | rs11584511 | 46892811 | T | C | 0.14 | 0.34 | 0.058 | 3.35E-09 |
| VEA | 1 | rs324422 | 46886782 | T | C | 0.23 | 0.27 | 0.047 | 4.66E-09 |
| VEA | 1 | rs10489770 | 46807597 | A | G | 0.14 | 0.32 | 0.058 | 4.22E-08 |
| VEA | 1 | rs72677586 | 46813848 | A | G | 0.14 | 0.32 | 0.058 | 4.22E-08 |
| VEA | 1 | rs10890392 | 46852061 | G | A | 0.14 | 0.32 | 0.058 | 4.22E-08 |
| sumEA | 1 | rs324420 | 46870761 | A | C | 0.21 | 0.32 | 0.053 | 1.36E-09 |
| sumEA | 1 | rs1571138 | 46895641 | A | G | 0.20 | 0.31 | 0.053 | 2.62E-09 |
| sumEA | 1 | rs324418 | 46872698 | G | A | 0.22 | 0.29 | 0.051 | 1.84E-08 |
| sumEA | 1 | rs324422 | 46886782 | T | C | 0.23 | 0.28 | 0.050 | 2.10E-08 |

| **B** |  |  |  |  |  |  |  |
| --- | --- | --- | --- | --- | --- | --- | --- |
| **SNPID** | **Associated**  **Gene ID** | **Associated**  **Transcript ID** | **Associated**  **Gene Name** | **Associated**  **Gene Type** | **Impact**  **Rating** | **Variant**  **Allele** | **Consequence**  **Terms** |
| rs324420 | ENSG00000117480 | ENST00000243167 | FAAH | Protein coding | MODERATE | A | Missense variant |
| rs324422 | (Intergenic) | (Intergenic) | (Intergenic) | (Intergenic) | MODIFIER | T | Intergenic variant |
| rs324418 | ENSG00000117480 | ENST00000243167 | FAAH | Protein coding | MODIFIER | G | Intron variant |
| rs1571138 | ENSG00000232022 | ENST00000446499 | FAAHP1 | pseudogene | MODIFIER | G | upstream |
| rs11584511 | ENSG00000232022 | ENST00000446499 | FAAHP1 | pseudogene | MODIFIER | T | upstream |
| rs10489770 | ENSG00000117481 | ENST00000307089 | NSUN4 | NMD | MODIFIER | A | intron_variant |
| rs72677586 | ENSG00000117481 | ENST00000307089 | NSUN4 | NMD | MODIFIER | A | intron_variant |
| rs10890392 | (Intergenic) | (Intergenic) | (Intergenic) | (Intergenic) | MODIFIER | G | intergenic_variant |

| **C** |  |  |  |  |  |  |
| --- | --- | --- | --- | --- | --- | --- |
| **SNPID** | **eQTL** | **Tissue** | **Other** | **GTEx whole blood** | **GWAS Catalog,**  **PhenoScanner** | **Gene Atlas** |
| rs324420 | FAAH | Multiple | FAAHP1, LURAP1, NSUN4, RAD54L, MKNK1 | FAAH, NSUN4 | X | X |
| rs324422 | FAAH | Multiple | FAAHP1, LURAP1, NSUN4, RAD54L, MKNK1, MOB3C | FAAH, NSUN4, MOB3C | X | X |
| rs324418 | FAAH | Multiple | FAAHP1, LURAP1, NSUN4, RAD54L, MKNK1 | FAAH, NSUN4 | X | X |
| rs1571138 | FAAH | Multiple | FAAHP1, LURAP1, NSUN4, RAD54L, MKNK1 | FAAH, NSUN4 | X | X |
| rs11584511 | FAAH | Multiple | FAAHP1, LURAP1, NSUN4, RAD54L, MKNK1, UQCRH | FAAH, NSUN4 | X | X |
| rs10489770 | FAAH | Multiple | FAAHP1, LURAP1, NSUN4, RAD54L, MKNK1, UQCRH | FAAH | X | X |
| rs72677586 | FAAH | Multiple | FAAHP1, LURAP1, NSUN4, RAD54L, MKNK1, UQCRH | FAAH | X | X |
| rs10890392 | FAAH | Multiple | FAAHP1, LURAP1, NSUN4, RAD54L, MKNK1, UQCRH | FAAH | X | X |

**Table S10.** Significant GWAS associations for CER species. Description of the GWAS significant associations identified.

| **Lipid** | **Chr** | **SNP** | **Position** | **A1** | **A2** | **MAF** | **Beta** | **SE** | **P-value** |
| --- | --- | --- | --- | --- | --- | --- | --- | --- | --- |
| N22S19 | 20 | rs438568 | 12958687 | A | G | 0.36 | 0.37 | 0.044 | 7.59E-18 |
| N22S19 | 20 | rs1321940 | 12959885 | A | G | 0.37 | 0.37 | 0.044 | 1.43E-17 |
| N22S19 | 20 | rs364585 | 12962718 | A | G | 0.37 | 0.37 | 0.044 | 1.43E-17 |
| N22S19 | 20 | rs168622 | 12966089 | T | G | 0.37 | 0.37 | 0.044 | 1.43E-17 |
| N22S19 | 20 | rs680379 | 12969400 | A | G | 0.37 | 0.37 | 0.043 | 2.91E-17 |
| N22S19 | 20 | rs686548 | 12973521 | A | T | 0.37 | 0.37 | 0.043 | 2.91E-17 |
| N22S19 | 20 | rs4814175 | 12959094 | A | T | 0.37 | 0.36 | 0.044 | 1.40E-16 |
| N22S19 | 20 | rs4814176 | 12959398 | T | C | 0.37 | 0.36 | 0.044 | 1.40E-16 |
| N22S19 | 20 | rs2327452 | 12952964 | A | C | 0.32 | 0.34 | 0.046 | 1.30E-13 |
| N22S19 | 20 | rs3848746 | 12950606 | A | G | 0.32 | 0.34 | 0.046 | 1.48E-13 |
| N22S19 | 20 | rs2327451 | 12953934 | C | A | 0.32 | 0.34 | 0.046 | 1.61E-13 |
| N22S19 | 20 | rs4508668 | 12955601 | T | C | 0.32 | 0.34 | 0.046 | 1.61E-13 |
| N22S19 | 20 | rs3903703 | 12945963 | A | G | 0.32 | 0.33 | 0.046 | 2.31E-13 |
| N22S19 | 20 | rs4814173 | 12947532 | G | C | 0.32 | 0.33 | 0.046 | 2.31E-13 |
| N22S19 | 20 | rs3848744 | 12942649 | A | G | 0.32 | 0.33 | 0.046 | 2.84E-13 |
| N22S19 | 20 | rs3843765 | 12943737 | G | A | 0.32 | 0.33 | 0.046 | 3.52E-13 |
| N22S19 | 20 | rs3848745 | 12944067 | G | A | 0.32 | 0.33 | 0.046 | 3.52E-13 |
| N22S19 | 20 | rs6041735 | 12940649 | T | C | 0.32 | 0.33 | 0.046 | 3.97E-13 |
| N22S19 | 20 | rs4813102 | 12947883 | A | T | 0.37 | 0.30 | 0.045 | 9.50E-12 |
| N23S20 | 20 | rs1321940 | 12959885 | A | G | 0.37 | 0.32 | 0.048 | 4.62E-11 |
| N23S20 | 20 | rs364585 | 12962718 | A | G | 0.37 | 0.32 | 0.048 | 4.62E-11 |
| N23S20 | 20 | rs168622 | 12966089 | T | G | 0.37 | 0.32 | 0.048 | 4.62E-11 |
| N23S20 | 20 | rs680379 | 12969400 | A | G | 0.37 | 0.32 | 0.048 | 5.14E-11 |
| N23S20 | 20 | rs686548 | 12973521 | A | T | 0.37 | 0.32 | 0.048 | 5.14E-11 |
| N23S20 | 20 | rs438568 | 12958687 | A | G | 0.37 | 0.31 | 0.048 | 7.07E-11 |
| N23S20 | 20 | rs4814175 | 12959094 | A | T | 0.37 | 0.31 | 0.048 | 2.07E-10 |
| N23S20 | 20 | rs4814176 | 12959398 | T | C | 0.37 | 0.31 | 0.048 | 2.07E-10 |
| N23S20 | 20 | rs2327451 | 12953934 | C | A | 0.32 | 0.28 | 0.050 | 1.50E-08 |
| N23S20 | 20 | rs4508668 | 12955601 | T | C | 0.32 | 0.28 | 0.050 | 1.50E-08 |
| N23S20 | 20 | rs2327452 | 12952964 | A | C | 0.32 | 0.28 | 0.050 | 1.70E-08 |
| N23S20 | 20 | rs3903703 | 12945963 | A | G | 0.32 | 0.28 | 0.050 | 1.72E-08 |
| N23S20 | 20 | rs4814173 | 12947532 | G | C | 0.32 | 0.28 | 0.050 | 1.72E-08 |
| N23S20 | 20 | rs3848746 | 12950606 | A | G | 0.32 | 0.28 | 0.050 | 2.28E-08 |
| N23S20 | 20 | rs4813102 | 12947883 | A | T | 0.37 | 0.27 | 0.049 | 2.90E-08 |
| N24DS19 | 20 | rs1321940 | 12959885 | A | G | 0.37 | 0.44 | 0.047 | 3.14E-21 |
| N24DS19 | 20 | rs364585 | 12962718 | A | G | 0.37 | 0.44 | 0.047 | 3.14E-21 |
| N24DS19 | 20 | rs168622 | 12966089 | T | G | 0.37 | 0.44 | 0.047 | 3.14E-21 |
| N24DS19 | 20 | rs438568 | 12958687 | A | G | 0.36 | 0.44 | 0.047 | 4.18E-21 |
| N24DS19 | 20 | rs680379 | 12969400 | A | G | 0.37 | 0.43 | 0.046 | 1.13E-20 |
| N24DS19 | 20 | rs686548 | 12973521 | A | T | 0.37 | 0.43 | 0.046 | 1.13E-20 |
| N24DS19 | 20 | rs4814175 | 12959094 | A | T | 0.37 | 0.43 | 0.047 | 1.85E-20 |
| N24DS19 | 20 | rs4814176 | 12959398 | T | C | 0.37 | 0.43 | 0.047 | 1.85E-20 |
| N24DS19 | 20 | rs3848746 | 12950606 | A | G | 0.32 | 0.41 | 0.049 | 2.37E-17 |
| N24DS19 | 20 | rs2327451 | 12953934 | C | A | 0.32 | 0.40 | 0.049 | 1.11E-16 |
| N24DS19 | 20 | rs4508668 | 12955601 | T | C | 0.32 | 0.40 | 0.049 | 1.11E-16 |
| N24DS19 | 20 | rs3903703 | 12945963 | A | G | 0.32 | 0.40 | 0.049 | 1.29E-16 |
| N24DS19 | 20 | rs4814173 | 12947532 | G | C | 0.32 | 0.40 | 0.049 | 1.29E-16 |
| N24DS19 | 20 | rs2327452 | 12952964 | A | C | 0.32 | 0.40 | 0.049 | 1.40E-16 |
| N24DS19 | 20 | rs3843765 | 12943737 | G | A | 0.32 | 0.40 | 0.049 | 2.55E-16 |
| N24DS19 | 20 | rs3848745 | 12944067 | G | A | 0.32 | 0.40 | 0.049 | 2.55E-16 |
| N24DS19 | 20 | rs6041735 | 12940649 | T | C | 0.32 | 0.40 | 0.049 | 2.86E-16 |
| N24DS19 | 20 | rs3848744 | 12942649 | A | G | 0.32 | 0.40 | 0.049 | 3.21E-16 |
| N24DS19 | 20 | rs4813102 | 12947883 | A | T | 0.37 | 0.34 | 0.047 | 4.74E-13 |
| N24DS19 | 20 | rs6078854 | 12960153 | A | T | 0.40 | -0.30 | 0.046 | 4.65E-11 |
| N24DS19 | 20 | rs4544513 | 12954215 | T | C | 0.35 | -0.31 | 0.047 | 5.46E-11 |
| N24DS19 | 20 | rs6109637 | 12954804 | T | C | 0.35 | -0.31 | 0.047 | 5.46E-11 |
| N24DS19 | 20 | rs382003 | 12963171 | A | G | 0.30 | -0.29 | 0.049 | 1.59E-09 |
| N24DS19 | 20 | rs360539 | 12966440 | G | T | 0.30 | -0.29 | 0.048 | 2.57E-09 |
| N24DS19 | 20 | rs73079703 | 12941782 | T | C | 0.30 | -0.29 | 0.049 | 3.06E-09 |
| N24DS19 | 20 | rs8183164 | 12942600 | A | C | 0.30 | -0.29 | 0.049 | 3.06E-09 |
| N24DS19 | 20 | rs6131414 | 12945669 | A | G | 0.30 | -0.29 | 0.049 | 3.06E-09 |
| N24DS19 | 20 | rs7272107 | 12946328 | A | G | 0.30 | -0.29 | 0.049 | 3.06E-09 |
| N24DS19 | 20 | rs73079713 | 12947141 | T | C | 0.30 | -0.29 | 0.049 | 3.06E-09 |
| N24DS19 | 20 | rs6134734 | 12952640 | T | A | 0.30 | -0.29 | 0.049 | 4.20E-09 |
| N24DS19 | 20 | rs6109634 | 12953314 | G | A | 0.30 | -0.29 | 0.049 | 4.20E-09 |
| N24DS19 | 20 | rs3848748 | 12957587 | C | G | 0.29 | -0.29 | 0.049 | 4.56E-09 |
| N24DS19 | 20 | rs3848749 | 12962089 | C | T | 0.29 | -0.29 | 0.049 | 4.56E-09 |
| N24DS19 | 20 | rs6131417 | 12967751 | A | G | 0.29 | -0.28 | 0.049 | 7.26E-09 |
| N24DS19 | 20 | rs6134740 | 12968649 | C | G | 0.29 | -0.28 | 0.049 | 7.26E-09 |
| N24DS19 | 20 | rs6134741 | 12970842 | A | G | 0.29 | -0.28 | 0.049 | 7.26E-09 |
| N24DS19 | 20 | rs59131252 | 12975257 | A | C | 0.29 | -0.27 | 0.049 | 2.02E-08 |
| N24DS20 | 20 | rs680379 | 12969400 | A | G | 0.37 | 0.30 | 0.047 | 1.48E-10 |
| N24DS20 | 20 | rs686548 | 12973521 | A | T | 0.37 | 0.30 | 0.047 | 1.48E-10 |
| N24DS20 | 20 | rs1321940 | 12959885 | A | G | 0.37 | 0.30 | 0.047 | 1.79E-10 |
| N24DS20 | 20 | rs364585 | 12962718 | A | G | 0.37 | 0.30 | 0.047 | 1.79E-10 |
| N24DS20 | 20 | rs168622 | 12966089 | T | G | 0.37 | 0.30 | 0.047 | 1.79E-10 |
| N24DS20 | 20 | rs438568 | 12958687 | A | G | 0.37 | 0.30 | 0.047 | 2.62E-10 |
| N24DS20 | 20 | rs4814175 | 12959094 | A | T | 0.37 | 0.30 | 0.047 | 2.89E-10 |
| N24DS20 | 20 | rs4814176 | 12959398 | T | C | 0.37 | 0.30 | 0.047 | 2.89E-10 |
| N24DS20 | 20 | rs3848746 | 12950606 | A | G | 0.32 | 0.28 | 0.050 | 1.88E-08 |
| N24DS20 | 20 | rs2327451 | 12953934 | C | A | 0.32 | 0.28 | 0.050 | 2.34E-08 |
| N24DS20 | 20 | rs4508668 | 12955601 | T | C | 0.32 | 0.28 | 0.050 | 2.34E-08 |
| N24DS20 | 20 | rs2327452 | 12952964 | A | C | 0.32 | 0.27 | 0.050 | 2.81E-08 |
| N24DS20 | 20 | rs3903703 | 12945963 | A | G | 0.32 | 0.27 | 0.050 | 3.01E-08 |
| N24DS20 | 20 | rs4814173 | 12947532 | G | C | 0.32 | 0.27 | 0.050 | 3.01E-08 |
| N24S16 | 14 | rs7160525 | 64232220 | A | G | 0.14 | 0.36 | 0.058 | 5.67E-10 |
| N24S16 | 14 | rs17101394 | 64232386 | A | G | 0.14 | 0.36 | 0.058 | 5.67E-10 |
| N24S16 | 14 | rs8008068 | 64233717 | G | A | 0.14 | 0.36 | 0.058 | 5.67E-10 |
| N24S16 | 14 | rs8008070 | 64233720 | T | A | 0.14 | 0.36 | 0.058 | 5.67E-10 |
| N24S16 | 14 | rs8012828 | 64233980 | T | C | 0.14 | 0.36 | 0.058 | 5.67E-10 |
| N24S16 | 14 | rs34609767 | 64234034 | G | T | 0.14 | 0.36 | 0.058 | 5.67E-10 |
| N24S16 | 14 | rs4902243 | 64234243 | G | A | 0.14 | 0.36 | 0.058 | 5.67E-10 |
| N24S16 | 14 | **rs7157785** | 64235556 | T | G | 0.14 | 0.36 | 0.058 | 5.67E-10 |
| N24S16 | 14 | rs34817779 | 64236003 | T | C | 0.14 | 0.36 | 0.058 | 5.67E-10 |
| N24S16 | 14 | rs35372182 | 64236157 | G | A | 0.14 | 0.36 | 0.058 | 5.67E-10 |
| N24S16 | 14 | rs12897637 | 64239351 | C | T | 0.14 | 0.36 | 0.058 | 5.67E-10 |
| N24S16 | 14 | rs12878001 | 64239629 | G | T | 0.14 | 0.36 | 0.058 | 5.67E-10 |
| N24S16 | 20 | rs3848746 | 12950606 | A | G | 0.32 | 0.27 | 0.045 | 1.63E-09 |
| N24S16 | 20 | rs2327452 | 12952964 | A | C | 0.32 | 0.27 | 0.045 | 2.33E-09 |
| N24S16 | 20 | rs2327451 | 12953934 | C | A | 0.32 | 0.26 | 0.045 | 3.63E-09 |
| N24S16 | 20 | rs4508668 | 12955601 | T | C | 0.32 | 0.26 | 0.045 | 3.63E-09 |
| N24S16 | 20 | rs3903703 | 12945963 | A | G | 0.32 | 0.26 | 0.045 | 3.92E-09 |
| N24S16 | 20 | rs4814173 | 12947532 | G | C | 0.32 | 0.26 | 0.045 | 3.92E-09 |
| N24S16 | 20 | rs6041735 | 12940649 | T | C | 0.32 | 0.26 | 0.045 | 4.35E-09 |
| N24S16 | 20 | rs438568 | 12958687 | A | G | 0.36 | 0.25 | 0.043 | 5.41E-09 |
| N24S16 | 20 | rs680379 | 12969400 | A | G | 0.37 | 0.25 | 0.043 | 5.76E-09 |
| N24S16 | 20 | rs686548 | 12973521 | A | T | 0.37 | 0.25 | 0.043 | 5.76E-09 |
| N24S16 | 20 | rs3848744 | 12942649 | A | G | 0.32 | 0.26 | 0.045 | 5.83E-09 |
| N24S16 | 20 | rs1321940 | 12959885 | A | G | 0.37 | 0.25 | 0.043 | 8.37E-09 |
| N24S16 | 20 | rs364585 | 12962718 | A | G | 0.37 | 0.25 | 0.043 | 8.37E-09 |
| N24S16 | 20 | rs168622 | 12966089 | T | G | 0.37 | 0.25 | 0.043 | 8.37E-09 |
| N24S16 | 20 | rs3843765 | 12943737 | G | A | 0.32 | 0.26 | 0.045 | 8.98E-09 |
| N24S16 | 20 | rs3848745 | 12944067 | G | A | 0.32 | 0.26 | 0.045 | 8.98E-09 |
| N24S16 | 20 | rs4814175 | 12959094 | A | T | 0.37 | 0.24 | 0.043 | 2.58E-08 |
| N24S16 | 20 | rs4814176 | 12959398 | T | C | 0.37 | 0.24 | 0.043 | 2.58E-08 |
| N24S19 | 20 | rs438568 | 12958687 | A | G | 0.37 | 0.47 | 0.043 | 1.00E-27 |
| N24S19 | 20 | rs1321940 | 12959885 | A | G | 0.37 | 0.47 | 0.043 | 1.92E-27 |
| N24S19 | 20 | rs364585 | 12962718 | A | G | 0.37 | 0.47 | 0.043 | 1.92E-27 |
| N24S19 | 20 | rs168622 | 12966089 | T | G | 0.37 | 0.47 | 0.043 | 1.92E-27 |
| N24S19 | 20 | rs680379 | 12969400 | A | G | 0.37 | 0.46 | 0.043 | 4.82E-27 |
| N24S19 | 20 | rs686548 | 12973521 | A | T | 0.37 | 0.46 | 0.043 | 4.82E-27 |
| N24S19 | 20 | rs4814175 | 12959094 | A | T | 0.37 | 0.45 | 0.043 | 5.64E-26 |
| N24S19 | 20 | rs4814176 | 12959398 | T | C | 0.37 | 0.45 | 0.043 | 5.64E-26 |
| N24S19 | 20 | rs3848746 | 12950606 | A | G | 0.33 | 0.43 | 0.045 | 5.81E-22 |
| N24S19 | 20 | rs2327452 | 12952964 | A | C | 0.32 | 0.43 | 0.045 | 7.61E-22 |
| N24S19 | 20 | rs2327451 | 12953934 | C | A | 0.32 | 0.43 | 0.045 | 1.05E-21 |
| N24S19 | 20 | rs4508668 | 12955601 | T | C | 0.32 | 0.43 | 0.045 | 1.05E-21 |
| N24S19 | 20 | rs3903703 | 12945963 | A | G | 0.32 | 0.43 | 0.045 | 1.32E-21 |
| N24S19 | 20 | rs4814173 | 12947532 | G | C | 0.32 | 0.43 | 0.045 | 1.32E-21 |
| N24S19 | 20 | rs6041735 | 12940649 | T | C | 0.32 | 0.42 | 0.045 | 9.75E-21 |
| N24S19 | 20 | rs3848744 | 12942649 | A | G | 0.32 | 0.42 | 0.045 | 1.04E-20 |
| N24S19 | 20 | rs3843765 | 12943737 | G | A | 0.32 | 0.42 | 0.045 | 1.42E-20 |
| N24S19 | 20 | rs3848745 | 12944067 | G | A | 0.32 | 0.42 | 0.045 | 1.42E-20 |
| N24S19 | 20 | rs4813102 | 12947883 | A | T | 0.37 | 0.38 | 0.044 | 5.60E-18 |
| N24S19 | 20 | rs608994 | 12980885 | G | A | 0.31 | 0.30 | 0.044 | 2.92E-11 |
| N24S19 | 20 | rs6078854 | 12960153 | A | T | 0.40 | -0.28 | 0.042 | 4.29E-11 |
| N24S19 | 20 | rs3910136 | 12962261 | A | T | 0.34 | -0.26 | 0.044 | 4.61E-09 |
| N24S19 | 20 | rs6041755 | 12973617 | T | C | 0.33 | -0.25 | 0.044 | 1.07E-08 |
| N24S19 | 20 | rs4544513 | 12954215 | T | C | 0.35 | -0.25 | 0.043 | 1.61E-08 |
| N24S19 | 20 | rs6109637 | 12954804 | T | C | 0.35 | -0.25 | 0.043 | 1.61E-08 |
| N24S19 | 20 | rs3848754 | 12971345 | C | T | 0.28 | -0.26 | 0.047 | 3.51E-08 |
| N24S19 | 20 | rs3848755 | 12971437 | C | T | 0.28 | -0.26 | 0.047 | 3.51E-08 |
| N24S19 | 20 | rs13037956 | 12974302 | A | C | 0.28 | -0.26 | 0.047 | 3.51E-08 |
| N24S19 | 20 | rs6074538 | 12974493 | T | C | 0.28 | -0.26 | 0.047 | 3.51E-08 |
| N24S19 | 20 | rs6078866 | 12974567 | G | A | 0.28 | -0.26 | 0.047 | 3.51E-08 |
| N24S19 | 20 | rs6074539 | 12974665 | A | G | 0.28 | -0.26 | 0.047 | 3.51E-08 |
| N24S20 | 20 | rs680379 | 12969400 | A | G | 0.37 | 0.38 | 0.049 | 9.86E-15 |
| N24S20 | 20 | rs686548 | 12973521 | A | T | 0.37 | 0.38 | 0.049 | 9.86E-15 |
| N24S20 | 20 | rs1321940 | 12959885 | A | G | 0.37 | 0.38 | 0.049 | 1.20E-14 |
| N24S20 | 20 | rs364585 | 12962718 | A | G | 0.37 | 0.38 | 0.049 | 1.20E-14 |
| N24S20 | 20 | rs168622 | 12966089 | T | G | 0.37 | 0.38 | 0.049 | 1.20E-14 |
| N24S20 | 20 | rs438568 | 12958687 | A | G | 0.37 | 0.38 | 0.049 | 2.59E-14 |
| N24S20 | 20 | rs4814175 | 12959094 | A | T | 0.37 | 0.37 | 0.050 | 5.79E-14 |
| N24S20 | 20 | rs4814176 | 12959398 | T | C | 0.37 | 0.37 | 0.050 | 5.79E-14 |
| N24S20 | 20 | rs2327451 | 12953934 | C | A | 0.33 | 0.34 | 0.052 | 4.27E-11 |
| N24S20 | 20 | rs4508668 | 12955601 | T | C | 0.33 | 0.34 | 0.052 | 4.27E-11 |
| N24S20 | 20 | rs3903703 | 12945963 | A | G | 0.33 | 0.34 | 0.052 | 5.46E-11 |
| N24S20 | 20 | rs4814173 | 12947532 | G | C | 0.33 | 0.34 | 0.052 | 5.46E-11 |
| N24S20 | 20 | rs2327452 | 12952964 | A | C | 0.33 | 0.34 | 0.052 | 5.60E-11 |
| N24S20 | 20 | rs3848746 | 12950606 | A | G | 0.33 | 0.34 | 0.052 | 6.22E-11 |
| N24S20 | 20 | rs3843765 | 12943737 | G | A | 0.32 | 0.33 | 0.052 | 1.22E-10 |
| N24S20 | 20 | rs3848745 | 12944067 | G | A | 0.32 | 0.33 | 0.052 | 1.22E-10 |
| N24S20 | 20 | rs3848744 | 12942649 | A | G | 0.32 | 0.33 | 0.052 | 1.59E-10 |
| N24S20 | 20 | rs6041735 | 12940649 | T | C | 0.32 | 0.33 | 0.052 | 3.08E-10 |
| N24S20 | 20 | rs4813102 | 12947883 | A | T | 0.38 | 0.30 | 0.050 | 1.44E-09 |
| N25S20 | 20 | rs680379 | 12969400 | A | G | 0.37 | 0.29 | 0.048 | 9.18E-10 |
| N25S20 | 20 | rs686548 | 12973521 | A | T | 0.37 | 0.29 | 0.048 | 9.18E-10 |
| N25S20 | 20 | rs1321940 | 12959885 | A | G | 0.37 | 0.29 | 0.048 | 1.10E-09 |
| N25S20 | 20 | rs364585 | 12962718 | A | G | 0.37 | 0.29 | 0.048 | 1.10E-09 |
| N25S20 | 20 | rs168622 | 12966089 | T | G | 0.37 | 0.29 | 0.048 | 1.10E-09 |
| N25S20 | 20 | rs438568 | 12958687 | A | G | 0.36 | 0.29 | 0.048 | 1.19E-09 |
| N25S20 | 20 | rs4814175 | 12959094 | A | T | 0.37 | 0.29 | 0.048 | 2.08E-09 |
| N25S20 | 20 | rs4814176 | 12959398 | T | C | 0.37 | 0.29 | 0.048 | 2.08E-09 |
| N26S19 | 20 | rs438568 | 12958687 | A | G | 0.36 | 0.33 | 0.045 | 3.40E-13 |
| N26S19 | 20 | rs1321940 | 12959885 | A | G | 0.36 | 0.32 | 0.045 | 3.98E-13 |
| N26S19 | 20 | rs364585 | 12962718 | A | G | 0.36 | 0.32 | 0.045 | 3.98E-13 |
| N26S19 | 20 | rs168622 | 12966089 | T | G | 0.36 | 0.32 | 0.045 | 3.98E-13 |
| N26S19 | 20 | rs680379 | 12969400 | A | G | 0.37 | 0.32 | 0.045 | 7.18E-13 |
| N26S19 | 20 | rs686548 | 12973521 | A | T | 0.37 | 0.32 | 0.045 | 7.18E-13 |
| N26S19 | 20 | rs4814175 | 12959094 | A | T | 0.37 | 0.32 | 0.045 | 1.14E-12 |
| N26S19 | 20 | rs4814176 | 12959398 | T | C | 0.37 | 0.32 | 0.045 | 1.14E-12 |
| N26S19 | 6 | rs6940658 | 14238511 | C | G | 0.09 | 0.48 | 0.075 | 1.10E-10 |
| N26S19 | 6 | rs4333409 | 14240330 | A | C | 0.09 | 0.48 | 0.075 | 1.10E-10 |
| N26S19 | 6 | rs2039310 | 14250304 | A | G | 0.09 | 0.48 | 0.075 | 1.10E-10 |
| N26S19 | 6 | rs9382948 | 14251751 | A | G | 0.09 | 0.48 | 0.075 | 1.10E-10 |
| N26S19 | 6 | rs6910045 | 14255808 | G | A | 0.09 | 0.48 | 0.075 | 1.10E-10 |
| N26S19 | 6 | rs9367828 | 14236554 | G | A | 0.09 | 0.48 | 0.075 | 2.00E-10 |
| N26S19 | 6 | **rs9370735** | 14244517 | T | G | 0.09 | 0.48 | 0.075 | 2.00E-10 |
| N26S19 | 6 | rs6940973 | 14238655 | C | T | 0.09 | 0.47 | 0.076 | 8.61E-10 |
| N26S19 | 6 | rs1537152 | 14240435 | C | A | 0.09 | 0.47 | 0.076 | 8.61E-10 |
| N26S19 | 6 | **rs1537151** | 14240594 | C | A | 0.09 | 0.47 | 0.076 | 8.61E-10 |
| N26S19 | 6 | rs9396477 | 14234971 | C | G | 0.08 | 0.44 | 0.078 | 1.54E-08 |
| N26S19 | 20 | rs3848746 | 12950606 | A | G | 0.32 | 0.26 | 0.047 | 1.97E-08 |
| N26S19 | 20 | rs6041735 | 12940649 | T | C | 0.32 | 0.26 | 0.047 | 2.00E-08 |
| N26S19 | 6 | rs12208698 | 14237070 | G | T | 0.07 | 0.46 | 0.082 | 2.07E-08 |
| N26S19 | 6 | rs12190393 | 14239216 | A | G | 0.07 | 0.46 | 0.082 | 2.07E-08 |
| N26S19 | 6 | rs12212956 | 14240006 | G | A | 0.07 | 0.46 | 0.082 | 2.07E-08 |
| N26S19 | 6 | **rs12207359** | 14244274 | T | G | 0.07 | 0.46 | 0.082 | 2.07E-08 |
| N26S19 | 6 | rs75762794 | 14245458 | C | G | 0.07 | 0.46 | 0.082 | 2.07E-08 |
| N26S19 | 6 | rs2876349 | 14246531 | T | C | 0.07 | 0.46 | 0.082 | 2.07E-08 |
| N26S19 | 6 | rs12213267 | 14247608 | C | G | 0.07 | 0.46 | 0.082 | 2.07E-08 |
| N26S19 | 6 | rs115366574 | 14250710 | C | T | 0.07 | 0.46 | 0.082 | 2.07E-08 |
| N26S19 | 6 | rs79263173 | 14253258 | T | A | 0.07 | 0.46 | 0.082 | 2.07E-08 |
| N26S19 | 20 | rs2327452 | 12952964 | A | C | 0.32 | 0.26 | 0.047 | 2.29E-08 |
| N26S19 | 20 | rs3848744 | 12942649 | A | G | 0.32 | 0.26 | 0.047 | 2.55E-08 |
| N26S19 | 20 | rs2327451 | 12953934 | C | A | 0.32 | 0.26 | 0.047 | 2.92E-08 |
| N26S19 | 20 | rs4508668 | 12955601 | T | C | 0.32 | 0.26 | 0.047 | 2.92E-08 |
| N26S19 | 20 | rs3843765 | 12943737 | G | A | 0.32 | 0.26 | 0.047 | 3.25E-08 |
| N26S19 | 20 | rs3848745 | 12944067 | G | A | 0.32 | 0.26 | 0.047 | 3.25E-08 |
| N26S19 | 20 | rs3903703 | 12945963 | A | G | 0.32 | 0.26 | 0.047 | 3.31E-08 |
| N26S19 | 20 | rs4814173 | 12947532 | G | C | 0.32 | 0.26 | 0.047 | 3.31E-08 |
| N24S19ratio | 1 | rs4653568 | 224396329 | A | G | 0.35 | 0.29 | 0.046 | 2.36E-10 |
| N24S19ratio | 1 | rs4654000 | 224396487 | C | T | 0.35 | 0.29 | 0.046 | 2.36E-10 |
| N24S19ratio | 1 | rs9793489 | 224397459 | T | A | 0.35 | 0.29 | 0.046 | 2.36E-10 |
| N24S19ratio | 1 | rs2011117 | 224398639 | C | T | 0.35 | 0.29 | 0.046 | 2.36E-10 |
| N24S19ratio | 1 | rs908801 | 224398817 | T | C | 0.35 | 0.29 | 0.046 | 2.36E-10 |
| N24S19ratio | 1 | rs6681673 | 224398910 | T | C | 0.35 | 0.29 | 0.046 | 2.36E-10 |
| N24S19ratio | 1 | rs4654003 | 224399383 | T | C | 0.35 | 0.29 | 0.046 | 2.36E-10 |
| N24S19ratio | 1 | rs6682292 | 224400175 | A | G | 0.35 | 0.29 | 0.046 | 2.36E-10 |
| N24S19ratio | 1 | rs12038372 | 224363881 | T | C | 0.41 | 0.26 | 0.045 | 1.30E-08 |
| N24S19ratio | 1 | rs6426143 | 224318875 | G | A | 0.40 | 0.25 | 0.045 | 2.15E-08 |
| N24S19ratio | 1 | rs6682551 | 224352258 | C | T | 0.39 | 0.25 | 0.045 | 2.42E-08 |
| N24S19ratio | 1 | **rs10799505** | 224351023 | T | C | 0.40 | 0.25 | 0.045 | 3.24E-08 |
| N24S19ratio | 1 | rs10916403 | 224352551 | C | A | 0.40 | 0.25 | 0.045 | 3.24E-08 |
| N24S19ratio | 1 | rs55664906 | 224355682 | A | C | 0.40 | 0.25 | 0.045 | 3.24E-08 |
| N24S19ratio | 1 | rs12076788 | 224355701 | C | A | 0.40 | 0.25 | 0.045 | 3.24E-08 |
| N24S19ratio | 1 | rs4653563 | 224314776 | A | C | 0.40 | 0.25 | 0.045 | 3.68E-08 |
| N24S19ratio | 1 | rs4653564 | 224314814 | C | T | 0.40 | 0.25 | 0.045 | 3.68E-08 |
| N24S19ratio | 1 | rs6426139 | 224315128 | A | G | 0.40 | 0.25 | 0.045 | 3.68E-08 |
| N24S19ratio | 1 | rs10753454 | 224316813 | C | T | 0.40 | 0.25 | 0.045 | 3.68E-08 |
| N24S19ratio | 1 | rs7542293 | 224316881 | A | G | 0.40 | 0.25 | 0.045 | 3.68E-08 |
| N24S19ratio | 1 | rs7542474 | 224317043 | A | C | 0.40 | 0.25 | 0.045 | 3.68E-08 |
| N24S19ratio | 1 | rs6698041 | 224317551 | G | A | 0.40 | 0.25 | 0.045 | 3.68E-08 |
| N24S19ratio | 1 | rs7519433 | 224319003 | T | C | 0.40 | 0.25 | 0.045 | 3.68E-08 |
| N24S19ratio | 1 | rs12730611 | 224319825 | G | C | 0.40 | 0.25 | 0.045 | 3.68E-08 |
| N24S19ratio | 1 | rs2014782 | 224321492 | T | C | 0.40 | 0.25 | 0.045 | 3.68E-08 |
| N24S19ratio | 1 | rs869945 | 224321637 | G | A | 0.40 | 0.25 | 0.045 | 3.68E-08 |
| N24S19ratio | 1 | rs6691405 | 224322879 | A | G | 0.40 | 0.25 | 0.045 | 3.68E-08 |
| N24S19ratio | 1 | rs6685783 | 224322942 | T | C | 0.40 | 0.25 | 0.045 | 3.68E-08 |
| N24S19ratio | 1 | rs10916355 | 224323548 | T | C | 0.40 | 0.25 | 0.045 | 3.68E-08 |
| N24S19ratio | 1 | rs66506223 | 224325436 | C | G | 0.40 | 0.25 | 0.045 | 3.68E-08 |
| N24S19ratio | 1 | rs61827699 | 224325460 | G | A | 0.40 | 0.25 | 0.045 | 3.68E-08 |
| N24S19ratio | 1 | rs10916367 | 224329969 | G | A | 0.40 | 0.25 | 0.045 | 4.33E-08 |
| N24S19ratio | 1 | rs4653986 | 224330818 | A | G | 0.40 | 0.25 | 0.045 | 4.33E-08 |
| N24S19ratio | 1 | rs7518839 | 224331779 | G | A | 0.40 | 0.25 | 0.045 | 4.33E-08 |
| N24S19ratio | 1 | rs13374070 | 224332078 | A | G | 0.40 | 0.25 | 0.045 | 4.33E-08 |
| N24S19ratio | 1 | rs55736782 | 224334072 | G | A | 0.40 | 0.25 | 0.045 | 4.33E-08 |
| N24S19ratio | 1 | rs997297 | 224335492 | A | T | 0.40 | 0.25 | 0.045 | 4.33E-08 |
| N24S19ratio | 1 | rs997296 | 224335497 | C | G | 0.40 | 0.25 | 0.045 | 4.33E-08 |
| N24S19ratio | 1 | rs7531891 | 224335956 | A | G | 0.40 | 0.25 | 0.045 | 4.33E-08 |
| N24S19ratio | 1 | rs1492694 | 224337153 | G | A | 0.40 | 0.25 | 0.045 | 4.33E-08 |
| N24S19ratio | 1 | rs4653991 | 224337208 | G | A | 0.40 | 0.25 | 0.045 | 4.33E-08 |
| N24S19ratio | 1 | rs7546235 | 224338017 | T | C | 0.40 | 0.25 | 0.045 | 4.33E-08 |
| N24S19ratio | 1 | rs10916371 | 224338162 | A | G | 0.40 | 0.25 | 0.045 | 4.33E-08 |
| N24S19ratio | 1 | rs7524705 | 224339000 | A | G | 0.40 | 0.25 | 0.045 | 4.33E-08 |
| N24S19ratio | 1 | **rs1826421** | 224339447 | A | G | 0.40 | 0.25 | 0.045 | 4.33E-08 |
| N24S19ratio | 1 | rs12563153 | 224339647 | A | G | 0.40 | 0.25 | 0.045 | 4.33E-08 |
| N24S19ratio | 1 | rs8328 | 224346959 | G | A | 0.40 | 0.25 | 0.045 | 4.33E-08 |
| N24S19ratio | 1 | rs7526252 | 224348818 | T | C | 0.40 | 0.25 | 0.045 | 4.33E-08 |
| S19Sum | 20 | rs438568 | 12958687 | A | G | 0.37 | 0.48 | 0.043 | 4.46E-29 |
| S19Sum | 20 | rs1321940 | 12959885 | A | G | 0.37 | 0.48 | 0.043 | 6.33E-29 |
| S19Sum | 20 | rs364585 | 12962718 | A | G | 0.37 | 0.48 | 0.043 | 6.33E-29 |
| S19Sum | 20 | rs168622 | 12966089 | T | G | 0.37 | 0.48 | 0.043 | 6.33E-29 |
| S19Sum | 20 | rs680379 | 12969400 | A | G | 0.37 | 0.48 | 0.043 | 2.19E-28 |
| S19Sum | 20 | rs686548 | 12973521 | A | T | 0.37 | 0.48 | 0.043 | 2.19E-28 |
| S19Sum | 20 | rs4814175 | 12959094 | A | T | 0.37 | 0.47 | 0.043 | 2.37E-27 |
| S19Sum | 20 | rs4814176 | 12959398 | T | C | 0.37 | 0.47 | 0.043 | 2.37E-27 |
| S19Sum | 20 | rs3848746 | 12950606 | A | G | 0.33 | 0.44 | 0.045 | 1.11E-22 |
| S19Sum | 20 | rs2327452 | 12952964 | A | C | 0.32 | 0.44 | 0.045 | 1.56E-22 |
| S19Sum | 20 | rs2327451 | 12953934 | C | A | 0.32 | 0.44 | 0.045 | 1.95E-22 |
| S19Sum | 20 | rs4508668 | 12955601 | T | C | 0.32 | 0.44 | 0.045 | 1.95E-22 |
| S19Sum | 20 | rs3903703 | 12945963 | A | G | 0.32 | 0.44 | 0.045 | 2.68E-22 |
| S19Sum | 20 | rs4814173 | 12947532 | G | C | 0.32 | 0.44 | 0.045 | 2.68E-22 |
| S19Sum | 20 | rs3848744 | 12942649 | A | G | 0.32 | 0.43 | 0.045 | 2.12E-21 |
| S19Sum | 20 | rs3843765 | 12943737 | G | A | 0.32 | 0.43 | 0.045 | 2.64E-21 |
| S19Sum | 20 | rs3848745 | 12944067 | G | A | 0.32 | 0.43 | 0.045 | 2.64E-21 |
| S19Sum | 20 | rs6041735 | 12940649 | T | C | 0.32 | 0.43 | 0.045 | 3.28E-21 |
| S19Sum | 20 | rs4813102 | 12947883 | A | T | 0.37 | 0.39 | 0.044 | 7.47E-19 |
| S19Sum | 20 | rs6078854 | 12960153 | A | T | 0.40 | -0.30 | 0.042 | 3.38E-12 |
| S19Sum | 20 | rs608994 | 12980885 | G | A | 0.31 | 0.30 | 0.045 | 1.70E-11 |
| S19Sum | 20 | rs4544513 | 12954215 | T | C | 0.35 | -0.26 | 0.044 | 1.79E-09 |
| S19Sum | 20 | rs6109637 | 12954804 | T | C | 0.35 | -0.26 | 0.044 | 1.79E-09 |
| S19Sum | 20 | rs382003 | 12963171 | A | G | 0.30 | -0.26 | 0.045 | 6.06E-09 |
| S19Sum | 20 | rs360539 | 12966440 | G | T | 0.30 | -0.26 | 0.045 | 7.23E-09 |
| S19Sum | 20 | rs3910136 | 12962261 | A | T | 0.34 | -0.25 | 0.044 | 8.71E-09 |
| S19Sum | 20 | rs73079703 | 12941782 | T | C | 0.30 | -0.25 | 0.045 | 2.40E-08 |
| S19Sum | 20 | rs8183164 | 12942600 | A | C | 0.30 | -0.25 | 0.045 | 2.40E-08 |
| S19Sum | 20 | rs6131414 | 12945669 | A | G | 0.30 | -0.25 | 0.045 | 2.40E-08 |
| S19Sum | 20 | rs7272107 | 12946328 | A | G | 0.30 | -0.25 | 0.045 | 2.40E-08 |
| S19Sum | 20 | rs73079713 | 12947141 | T | C | 0.30 | -0.25 | 0.045 | 2.40E-08 |
| S19Sum | 20 | rs6041755 | 12973617 | T | C | 0.33 | -0.25 | 0.044 | 2.42E-08 |
| S19Sum | 20 | rs6134734 | 12952640 | T | A | 0.30 | -0.25 | 0.045 | 3.94E-08 |
| S19Sum | 20 | rs6109634 | 12953314 | G | A | 0.30 | -0.25 | 0.045 | 3.94E-08 |
| S19Sum | 20 | rs3848748 | 12957587 | C | G | 0.29 | -0.25 | 0.045 | 4.26E-08 |
| S19Sum | 20 | rs3848749 | 12962089 | C | T | 0.29 | -0.25 | 0.045 | 4.26E-08 |
| S19Sum | 20 | rs3848754 | 12971345 | C | T | 0.28 | -0.26 | 0.047 | 4.90E-08 |
| S19Sum | 20 | rs3848755 | 12971437 | C | T | 0.28 | -0.26 | 0.047 | 4.90E-08 |
| S19Sum | 20 | rs13037956 | 12974302 | A | C | 0.28 | -0.26 | 0.047 | 4.90E-08 |
| S19Sum | 20 | rs6074538 | 12974493 | T | C | 0.28 | -0.26 | 0.047 | 4.90E-08 |
| S19Sum | 20 | rs6078866 | 12974567 | G | A | 0.28 | -0.26 | 0.047 | 4.90E-08 |
| S19Sum | 20 | rs6074539 | 12974665 | A | G | 0.28 | -0.26 | 0.047 | 4.90E-08 |
| S20Sum | 20 | rs680379 | 12969400 | A | G | 0.37 | 0.40 | 0.050 | 5.17E-16 |
| S20Sum | 20 | rs686548 | 12973521 | A | T | 0.37 | 0.40 | 0.050 | 5.17E-16 |
| S20Sum | 20 | rs1321940 | 12959885 | A | G | 0.37 | 0.40 | 0.050 | 5.94E-16 |
| S20Sum | 20 | rs364585 | 12962718 | A | G | 0.37 | 0.40 | 0.050 | 5.94E-16 |
| S20Sum | 20 | rs168622 | 12966089 | T | G | 0.37 | 0.40 | 0.050 | 5.94E-16 |
| S20Sum | 20 | rs438568 | 12958687 | A | G | 0.37 | 0.40 | 0.050 | 1.12E-15 |
| S20Sum | 20 | rs4814175 | 12959094 | A | T | 0.37 | 0.39 | 0.050 | 3.56E-15 |
| S20Sum | 20 | rs4814176 | 12959398 | T | C | 0.37 | 0.39 | 0.050 | 3.56E-15 |
| S20Sum | 20 | rs2327451 | 12953934 | C | A | 0.33 | 0.35 | 0.052 | 1.31E-11 |
| S20Sum | 20 | rs4508668 | 12955601 | T | C | 0.33 | 0.35 | 0.052 | 1.31E-11 |
| S20Sum | 20 | rs2327452 | 12952964 | A | C | 0.32 | 0.35 | 0.052 | 1.47E-11 |
| S20Sum | 20 | rs3903703 | 12945963 | A | G | 0.32 | 0.35 | 0.052 | 1.74E-11 |
| S20Sum | 20 | rs4814173 | 12947532 | G | C | 0.32 | 0.35 | 0.052 | 1.74E-11 |
| S20Sum | 20 | rs3848746 | 12950606 | A | G | 0.33 | 0.35 | 0.052 | 1.77E-11 |
| S20Sum | 20 | rs3843765 | 12943737 | G | A | 0.32 | 0.34 | 0.052 | 4.04E-11 |
| S20Sum | 20 | rs3848745 | 12944067 | G | A | 0.32 | 0.34 | 0.052 | 4.04E-11 |
| S20Sum | 20 | rs3848744 | 12942649 | A | G | 0.32 | 0.34 | 0.052 | 4.52E-11 |
| S20Sum | 20 | rs6041735 | 12940649 | T | C | 0.32 | 0.34 | 0.052 | 8.35E-11 |
| S20Sum | 20 | rs4813102 | 12947883 | A | T | 0.38 | 0.32 | 0.051 | 3.91E-10 |

Table S11(A): Ensembl and GTEx summary of the significant SNPs identified by GWAS association for ceramides and related sphingolipid species. Description of the SNPs using the Ensembl API Client and summary of the information on eQTL status as identified using the GTEX browser.

| SNP ID | Gene Name | Gene Type | Variant Allele | Consequence Terms | GTEx | Liver or whole blood? | Other |
| --- | --- | --- | --- | --- | --- | --- | --- |
| rs438568 | LINC01723 | lncRNA | G | intron | SPTLC3 | Liver | X |
| rs1321940 | LINC01723 | lncRNA | G | intron | SPTLC3 | Liver | ISM1 (pancreas) |
| rs364585 | LINC01723 | lncRNA | G | downstream | SPTLC3 | Liver | X |
| rs168622 | LINC01723 | lncRNA | G | intron | SPTLC3 | Liver | X |
| rs680379 | LINC01723 | lncRNA | G | intron | SPTLC3 | Liver | X |
| rs686548 | LINC01723 | lncRNA | T | intron | SPTLC3 | Liver | ISM1 (pancreas) |
| rs4814175 | LINC01723 | lncRNA | T | intron | SPTLC3 | Liver | X |
| rs4814176 | LINC01723 | lncRNA | C | intron | SPTLC3 | Liver | X |
| rs2327452 | LINC01723 | lncRNA | C | intron | SPTLC3 | Liver | X |
| rs3848746 | LINC01723 | lncRNA | G | intron | SPTLC3 | Liver | X |
| rs2327451 | LINC01723 | lncRNA | A | intron | SPTLC3 | Liver | X |
| rs4508668 | LINC01723 | lncRNA | C | intron | SPTLC3 | Liver | X |
| rs3903703 | LINC01723 | lncRNA | G | intron | SPTLC3 | Liver | X |
| rs4814173 | LINC01723 | lncRNA | A | intron | SPTLC3 | Liver | X |
| rs3848744 | LINC01723 | lncRNA | G | intron | SPTLC3 | Liver | X |
| rs3843765 | LINC01723 | lncRNA | A | intron | SPTLC3 | Liver | X |
| rs3848745 | LINC01723 | lncRNA | A | intron | SPTLC3 | Liver | X |
| rs6041735 | LINC01723 | lncRNA | C | intron | SPTLC3 | Liver | X |
| rs4813102 | LINC01723 | lncRNA | T | intron | SPTLC3 | Liver | X |
| rs6078854 | LINC01723 | lncRNA | A | downstream | SPTLC3 | X | X |
| rs4544513 | LINC01723 | lncRNA | T | intron | SPTLC3 | Liver | X |
| rs6109637 | LINC01723 | lncRNA | T | intron | SPTLC3 | Liver | X |
| rs382003 | LINC01723 | lncRNA | A | downstream | SPTLC3 | Liver | X |
| rs360539 | LINC01723 | lncRNA | C | intron | SPTLC3 | Liver | X |
| rs73079703 | LINC01723 | lncRNA | T | intron | X | X | X |
| rs8183164 | LINC01723 | lncRNA | A | intron | X | X | X |
| rs6131414 | LINC01723 | lncRNA | A | intron | X | X | X |
| rs7272107 | LINC01723 | lncRNA | A | intron | X | X | X |
| rs73079713 | LINC01723 | lncRNA | T | intron | X | X | X |
| rs6134734 | LINC01723 | lncRNA | T | intron | X | X | X |
| rs6109634 | LINC01723 | lncRNA | G | intron | X | X | X |
| rs3848748 | LINC01723 | lncRNA | C | intron | X | X | X |
| rs3848749 | LINC01723 | lncRNA | C | downstream | X | X | X |
| rs6131417 | LINC01723 | lncRNA | A | intron | X | X | X |
| rs6134740 | LINC01723 | lncRNA | C | intron | X | X | X |
| rs6134741 | LINC01723 | lncRNA | A | intron | X | X | X |
| rs59131252 | LINC01723 | lncRNA | A | intron | X | X | X |
| rs7160525 | AL161670.1 | pseudogene | A | downstream | X | X | X |
| rs17101394 | AL161670.1 | pseudogene | A | downstream | X | X | X |
| rs8008068 | AL161670.1 | pseudogene | G | downstream | X | X | X |
| rs8008070 | AL161670.1 | pseudogene | T | downstream | X | X | X |
| rs8012828 | Intergenic | Intergenic | T | intergenic | X | X | X |
| rs34609767 | Intergenic | Intergenic | G | intergenic | X | X | X |
| rs4902243 | Intergenic | Intergenic | G | intergenic | X | X | X |
| rs7157785 | Intergenic | Intergenic | T | intergenic | X | X | X |
| rs34817779 | Intergenic | Intergenic | T | intergenic | X | X | X |
| rs35372182 | Intergenic | Intergenic | G | intergenic | X | X | X |
| rs12897637 | Intergenic | Intergenic | C | intergenic | X | X | X |
| rs12878001 | Intergenic | Intergenic | G | intergenic | X | X | X |
| rs608994 | LINC01723 | lncRNA | A | intron | SPTLC3 | Liver | X |
| rs3910136 | LINC01723 | lncRNA | A | downstream | NA | NA | NA |
| rs6041755 | LINC01723 | lncRNA | T | intron | SPTLC3 | X | X |
| rs3848754 | LINC01723 | lncRNA | C | intron | SPTLC3 | X | X |
| rs3848755 | LINC01723 | lncRNA | C | intron | X | X | X |
| rs13037956 | LINC01723 | lncRNA | A | intron | SPTLC3 | X | X |
| rs6074538 | LINC01723 | lncRNA | T | intron | SPTLC3 | X | X |
| rs6078866 | LINC01723 | lncRNA | G | intron | SPTLC3 | X | X |
| rs6074539 | LINC01723 | lncRNA | A | intron | X | X | X |
| rs6940658 | Intergenic | Intergenic | G | intergenic | X | X | X |
| rs4333409 | Intergenic | Intergenic | C | intergenic | X | X | X |
| rs2039310 | Intergenic | Intergenic | C | intergenic | X | X | X |
| rs9382948 | Intergenic | Intergenic | G | intergenic | X | X | X |
| rs6910045 | Intergenic | Intergenic | A | intergenic | RP3-500L14.2 | X | X |
| rs9367828 | AL353152.1 | lncRNA | A | upstream | X | X | X |
| rs9370735 | Intergenic | Intergenic | G | intergenic | X | X | X |
| rs6940973 | Intergenic | Intergenic | T | intergenic | X | X | X |
| rs1537152 | Intergenic | Intergenic | A | intergenic | X | X | X |
| rs1537151 | Intergenic | Intergenic | A | intergenic | X | X | X |
| rs9396477 | AL353152.1 | lncRNA | G | upstream | X | X | X |
| rs12208698 | Intergenic | Intergenic | A | intergenic | X | X | X |
| rs12190393 | Intergenic | Intergenic | A | intergenic | X | X | X |
| rs12212956 | Intergenic | Intergenic | G | intergenic | X | X | X |
| rs12207359 | Intergenic | Intergenic | T | intergenic | X | X | X |
| rs75762794 | Intergenic | Intergenic | C | intergenic | X | X | X |
| rs2876349 | Intergenic | Intergenic | A | intergenic | X | X | X |
| rs12213267 | Intergenic | Intergenic | C | intergenic | X | X | X |
| rs115366574 | Intergenic | Intergenic | C | intergenic | X | X | X |
| rs79263173 | Intergenic | Intergenic | T | intergenic | X | X | X |
| rs4653568 | AC092809.2 | lncRNA | G | downstream | FBXO28 | blood | DEGS1, RP11-365O16.3, CAPN8 |
| rs4654000 | AC092809.2 | lncRNA | G | exon | FBXO28 | blood | DEGS1, RP11-365O16.3, CAPN8, GTP2IP20 |
| rs9793489 | AC092809.2 | lncRNA | A | intron | FBXO28 | blood | DEGS1, RP11-365O16.3, CAPN8, GTP2IP20 |
| rs2011117 | AC092809.2 | lncRNA | T | intron | FBXO28 | blood | DEGS1, RP11-365O16.3, CAPN8, GTP2IP20 |
| rs908801 | AC092809.2 | lncRNA | A | intron | FBXO28 | blood | DEGS1, RP11-365O16.3, CAPN8, GTP2IP20 |
| rs6681673 | AC092809.2 | lncRNA | C | intron | FBXO28 | blood | DEGS1, RP11-365O16.3, CAPN8, GTP2IP20 |
| rs4654003 | AC092809.2 | lncRNA | C | intron | FBXO28 | blood | DEGS1, RP11-365O16.3, CAPN8, GTP2IP20 |
| rs6682292 | AC092809.2 | lncRNA | G | intron | FBXO28 | blood | DEGS1, RP11-365O16.3, CAPN8, GTP2IP20 |
| rs12038372 | DEGS1 | protein_coding | G | intron | FBXO28 | blood | DEGS1, RP11-365O16.3, CAPN8 |
| rs6426143 | FBXO28 | protein_coding | A | intron | FBXO28 | blood | DEGS1, RP11-365O16.3, CAPN8 |
| rs6682551 | FBXO28 | protein_coding | C | downstream | FBXO28 | blood | RP11-365O16.3 |
| rs10799505 | FBXO28 | protein_coding | T | downstream | FBXO28 | blood | DEGS1, RP11-365O16.3, CAPN8, CNIH3 |
| rs10916403 | FBXO28 | protein_coding | C | downstream | FBXO28 | blood | DEGS1, RP11-365O16.3, CAPN8, CNIH3 |
| rs55664906 | Intergenic | Intergenic | A | intergenic | FBXO28 | blood | DEGS1, RP11-365O16.3, CAPN8 |
| rs12076788 | Intergenic | Intergenic | A | intergenic | FBXO28 | blood | DEGS1, RP11-365O16.3, CAPN8 |
| rs4653563 | FBXO28 | protein_coding | C | intron | FBXO28 | blood | DEGS1, RP11-365O16.3, CAPN8 |
| rs4653564 | FBXO28 | protein_coding | T | intron | FBXO28 | blood | DEGS1, RP11-365O16.3, CAPN8 |
| rs6426139 | FBXO28 | protein_coding | G | intron | FBXO28 | blood | DEGS1, RP11-365O16.3, CAPN8 |
| rs10753454 | FBXO28 | protein_coding | G | intron | FBXO28 | blood | DEGS1, RP11-365O16.3, CAPN8 |
| rs7542293 | FBXO28 | protein_coding | G | intron | FBXO28 | blood | DEGS1, RP11-365O16.3, CAPN8 |
| rs7542474 | FBXO28 | protein_coding | C | intron | FBXO28 | blood | DEGS1, RP11-365O16.3, CAPN8 |
| rs6698041 | FBXO28 | protein_coding | A | intron | FBXO28 | blood | DEGS1, RP11-365O16.3, CAPN8 |
| rs7519433 | FBXO28 | protein_coding | T | intron | FBXO28 | blood | RP11-365O16.3 |
| rs12730611 | FBXO28 | protein_coding | C | intron | FBXO28 | blood | DEGS1, RP11-365O16.3, CAPN8 |
| rs2014782 | FBXO28 | protein_coding | T | intron | FBXO28 | blood | DEGS1, RP11-365O16.3, CAPN8 |
| rs869945 | FBXO28 | protein_coding | G | intron | FBXO28 | blood | DEGS1, RP11-365O16.3, CAPN8 |
| rs6691405 | FBXO28 | protein_coding | G | intron | FBXO28 | blood | DEGS1, RP11-365O16.3, CAPN8 |
| rs6685783 | FBXO28 | protein_coding | C | intron | FBXO28 | blood | DEGS1, RP11-365O16.3, CAPN8 |
| rs10916355 | FBXO28 | protein_coding | T | intron | FBXO28 | blood | DEGS1, RP11-365O16.3, CAPN8 |
| rs66506223 | FBXO28 | protein_coding | G | intron | FBXO28 | blood | DEGS1, RP11-365O16.3, CAPN8 |
| rs61827699 | FBXO28 | protein_coding | A | intron | FBXO28 | blood | DEGS1, RP11-365O16.3, CAPN8 |
| rs10916367 | FBXO28 | protein_coding | A | intron | FBXO28 | blood | DEGS1, RP11-365O16.3, CAPN8 |
| rs4653986 | FBXO28 | protein_coding | G | intron | FBXO28 | blood | DEGS1, RP11-365O16.3, CAPN8 |
| rs7518839 | FBXO28 | protein_coding | A | intron | FBXO28 | blood | DEGS1, RP11-365O16.3, CAPN8 |
| rs13374070 | FBXO28 | protein_coding | A | intron | FBXO28 | blood | DEGS1, RP11-365O16.3, CAPN8 |
| rs55736782 | FBXO28 | protein_coding | G | intron | FBXO28 | blood | DEGS1, RP11-365O16.3, CAPN8 |
| rs997297 | FBXO28 | protein_coding | T | intron | FBXO28 | blood | DEGS1, RP11-365O16.3, CAPN8 |
| rs997296 | FBXO28 | protein_coding | A | intron | FBXO28 | blood | DEGS1, RP11-365O16.3, CAPN8 |
| rs7531891 | FBXO28 | protein_coding | A | intron | FBXO28 | blood | DEGS1, RP11-365O16.3, CAPN8 |
| rs1492694 | FBXO28 | protein_coding | G | intron | FBXO28 | blood | DEGS1, RP11-365O16.3, CAPN8 |
| rs4653991 | FBXO28 | protein_coding | A | intron | FBXO28 | blood | DEGS1, RP11-365O16.3, CAPN8 |
| rs7546235 | FBXO28 | protein_coding | T | intron | FBXO28 | blood | DEGS1, RP11-365O16.3, CAPN8 |
| rs10916371 | FBXO28 | protein_coding | G | intron | FBXO28 | blood | DEGS1, RP11-365O16.3, CAPN8 |
| rs7524705 | FBXO28 | protein_coding | A | intron | FBXO28 | blood | DEGS1, RP11-365O16.3, CAPN8 |
| rs1826421 | FBXO28 | protein_coding | G | intron | FBXO28 | blood | DEGS1, RP11-365O16.3, CAPN8 |
| rs12563153 | FBXO28 | protein_coding | A | intron | FBXO28 | blood | DEGS1, RP11-365O16.3, CAPN8 |
| rs8328 | FBXO28 | protein_coding | A | 3_prime_UTR | FBXO28 | blood | DEGS1, RP11-365O16.3, CAPN8 |
| rs7526252 | FBXO28 | protein_coding | C | 3_prime_UTR | FBXO28 | blood | DEGS1, RP11-365O16.3, CAPN8 |

Table S11(B): GWAS Catalog and PhenoScanner searches, Gene Atlas PheWAS, and UCSC Genome Browser summaries of the significant SNPs identified by GWAS association for ceramides and related sphingolipid species. The table depicts the results of GWAS Catalog and PhenoScanner searches, Gene Atlas PheWAS, and known CER metabolic enzymes genes identified at that locus using UCSC Genome Browser.

| **SNP ID** | **GWAS Catalog, PhenoScanner** | **Gene Atlas** | **UCSC** |
| --- | --- | --- | --- |
| rs438568 | X | X | SPTLC3 |
| rs1321940 | X | X | SPTLC3 |
| rs364585 | LDL cholesterol, Arachidic acid 20:0, Lignoceric acid 24:0 | X | SPTLC3 |
| rs168622 | LDL cholesterol, Arachidic acid 20:0 | X | SPTLC3 |
| rs680379 | Sphingolipid levels, fatty acid levels, LDL cholesterol, Glycerophospholipid levels, Arachidic acid 20:0, ceramides | X | SPTLC3 |
| rs686548 | Serum metabolite ratios in chronic kidney disease | X | SPTLC3 |
| rs4814175 | X | X | SPTLC3 |
| rs4814176 | Sphingolipid levels, blood metabolites, Lignoceric acid 24:0, Arachidic acid 20:0, LDL cholesterol | X | SPTLC3 |
| rs2327452 | X | X | SPTLC3 |
| rs3848746 | X | X | SPTLC3 |
| rs2327451 | Lignoceric acid 24:0, Arachidic acid 20:0 | X | SPTLC3 |
| rs4508668 | Lignoceric acid 24:0, Arachidic acid 20:0 | X | SPTLC3 |
| rs3903703 | Fatty acids, Lignoceric acid 24:0, Arachidic acid 20:0 | X | SPTLC3 |
| rs4814173 | X | X | SPTLC3 |
| rs3848744 | X | X | SPTLC3 |
| rs3843765 | X | X | SPTLC3 |
| rs3848745 | X | X | SPTLC3 |
| rs6041735 | X | X | SPTLC3 |
| rs4813102 | X | X | SPTLC3 |
| rs6078854 | Arachidic acid 20:0 | X | SPTLC3 |
| rs4544513 | X | X | SPTLC3 |
| rs6109637 | X | X | SPTLC3 |
| rs382003 | X | X | SPTLC3 |
| rs360539 | X | X | SPTLC3 |
| rs73079703 | X | X | SPTLC3 |
| rs8183164 | X | X | SPTLC3 |
| rs6131414 | X | X | SPTLC3 |
| rs7272107 | X | X | SPTLC3 |
| rs73079713 | X | X | SPTLC3 |
| rs6134734 | X | X | SPTLC3 |
| rs6109634 | X | X | SPTLC3 |
| rs3848748 | X | X | SPTLC3 |
| rs3848749 | X | X | SPTLC3 |
| rs6131417 | X | X | SPTLC3 |
| rs6134740 | X | X | SPTLC3 |
| rs6134741 | X | X | SPTLC3 |
| rs59131252 | X | X | SPTLC3 |
| rs7160525 | Serum metabolite concentrations in chronic kidney disease, red cell distribution width, mean platelet volume, high light scatter reticulocyte percentage of red cells, reticulocyte fraction of red cells | Mean platelet (thrombocyte) volume (P=3.2825e-29); Red blood cell (erythrocyte) distribution width (P=5.963e-14); Platelet count (P=5.5601e-13); High light scatter reticulocyte percentage (P=1.2661e-12); High light scatter reticulocyte count (P=6.6865e-11); Immature reticulocyte fraction (P=1.0614e-10); Reticulocyte percentage (P=1.9119e-08) | SGPP1 |
| rs17101394 | Sphingolipid levels, red cell distribution width, mean platelet volume, high light scatter reticulocyte percentage of red cells, reticulocyte fraction of red cells, high light scatter reticulocyte count | Mean platelet (thrombocyte) volume (P=5.1305e-29); Red blood cell (erythrocyte) distribution width (P=4.9311e-14); Platelet count (P=6.397e-13); High light scatter reticulocyte percentage (P=3.2699e-12); High light scatter reticulocyte count (P=1.1051e-10); Immature reticulocyte fraction (P=2.0931e-10); Reticulocyte percentage (P=1.8191e-08) | SGPP1 |
| rs8008068 | Red cell distribution width, mean platelet volume, high light scatter reticulocyte percentage of red cells, reticulocyte fraction of red cells, high light scatter reticulocyte count | Mean platelet (thrombocyte) volume (P=4.7089e-29); Red blood cell (erythrocyte) distribution width (P=4.8152e-14); Platelet count (P=6.2582e-13); High light scatter reticulocyte percentage (P=1.866e-12); Immature reticulocyte fraction (P=5.3687e-11); High light scatter reticulocyte count (P=6.6258e-11); Reticulocyte percentage (P=2.0003e-08) | SGPP1 |
| rs8008070 | Serum metabolite ratios in chronic kidney disease, red cell distribution width, mean platelet volume, high light scatter reticulocyte percentage of red cells, reticulocyte fraction of red cells, high light scatter reticulocyte count | Mean platelet (thrombocyte) volume (P=2.8823e-29); Red blood cell (erythrocyte) distribution width (P=4.8987e-14); Platelet count (P=5.7716e-13); High light scatter reticulocyte percentage (P=1.7608e-12); Immature reticulocyte fraction (P=4.8545e-11); High light scatter reticulocyte count (P=6.1781e-11); Reticulocyte percentage (P=1.9361e-08) | SGPP1 |
| rs8012828 | Red cell distribution width, mean platelet volume, high light scatter reticulocyte percentage of red cells, reticulocyte fraction of red cells, high light scatter reticulocyte count | Mean platelet (thrombocyte) volume (P=4.6195e-29); Red blood cell (erythrocyte) distribution width (P=5.5573e-14); Platelet count (P=7.3757e-13); High light scatter reticulocyte percentage (P=3.4938e-12); High light scatter reticulocyte count (P=1.2452e-10); Immature reticulocyte fraction (P=2.1356e-10); Reticulocyte percentage (P=1.6812e-08) | SGPP1 |
| rs34609767 | Red cell distribution width, mean platelet volume, high light scatter reticulocyte percentage of red cells, reticulocyte fraction of red cells, high light scatter reticulocyte count | Mean platelet (thrombocyte) volume (P=3.1253e-29); Red blood cell (erythrocyte) distribution width (P=4.292e-14); Platelet count (P=5.6134e-13); High light scatter reticulocyte percentage (P=1.75e-12); High light scatter reticulocyte count (P=6.6063e-11); Immature reticulocyte fraction (P=6.8857e-11); Reticulocyte percentage (P=1.6786e-08) | SGPP1 |
| rs4902243 | Blood metabolite levels, Red cell distribution width, mean platelet volume, high light scatter reticulocyte percentage of red cells, reticulocyte fraction of red cells, high light scatter reticulocyte count | Mean platelet (thrombocyte) volume (P=4.2606e-29); Red blood cell (erythrocyte) distribution width (P=2.0997e-14); Platelet count (P=6.7994e-13); High light scatter reticulocyte percentage (P=2.5783e-12); High light scatter reticulocyte count (P=9.5096e-11); Immature reticulocyte fraction (P=1.0739e-10); Reticulocyte percentage (P=2.0268e-08) | SGPP1 |
| rs7157785 | Sphingolipid levels, blood metabolites, glycerophospholipids, total cholesterol, red cell distribution width, mean platelet volume, high light scatter reticulocyte percentage of red cells, reticulocyte fraction of red cells, high light scatter reticulocyte count | Mean platelet (thrombocyte) volume (P=1.2992e-28); Red blood cell (erythrocyte) distribution width (P=3.5732e-13); Platelet count (P=9.5427e-13); High light scatter reticulocyte percentage (P=1.0509e-12); Immature reticulocyte fraction (P=5.289e-11); High light scatter reticulocyte count (P=5.6669e-11); Reticulocyte percentage (P=1.6633e-08) | SGPP1 |
| rs34817779 | Red cell distribution width, mean platelet volume, high light scatter reticulocyte percentage of red cells, reticulocyte fraction of red cells, high light scatter reticulocyte count | Mean platelet (thrombocyte) volume (P=3.6761e-29); Red blood cell (erythrocyte) distribution width (P=3.6016e-14); Platelet count (P=4.2642e-13); High light scatter reticulocyte percentage (P=1.5459e-12); High light scatter reticulocyte count (P=6.0295e-11); Immature reticulocyte fraction (P=8.1684e-11); Reticulocyte percentage (P=1.5888e-08) | SGPP1 |
| rs35372182 | Red cell distribution width, mean platelet volume, high light scatter reticulocyte percentage of red cells, reticulocyte fraction of red cells, high light scatter reticulocyte count | Mean platelet (thrombocyte) volume (P=4.1731e-29); Red blood cell (erythrocyte) distribution width (P=4.1885e-14); Platelet count (P=4.8832e-13); High light scatter reticulocyte percentage (P=2.0206e-12); High light scatter reticulocyte count (P=7.8142e-11); Immature reticulocyte fraction (P=8.981e-11); Reticulocyte percentage (P=1.7224e-08) | SGPP1 |
| rs12897637 | Red cell distribution width, mean platelet volume, high light scatter reticulocyte percentage of red cells, reticulocyte fraction of red cells, high light scatter reticulocyte count | Mean platelet (thrombocyte) volume (P=9.1577e-29); Red blood cell (erythrocyte) distribution width (P=1.2123e-14); Platelet count (P=3.9706e-13); High light scatter reticulocyte percentage (P=1.2183e-12); High light scatter reticulocyte count (P=4.5639e-11); Immature reticulocyte fraction (P=9.9928e-11); Reticulocyte percentage (P=1.2307e-08) | SGPP1 |
| rs12878001 | Red cell distribution width, mean platelet volume, high light scatter reticulocyte percentage of red cells, reticulocyte fraction of red cells, high light scatter reticulocyte count | Mean platelet (thrombocyte) volume (P=4.1485e-29); Red blood cell (erythrocyte) distribution width (P=1.9038e-14); Platelet count (P=2.7341e-13); High light scatter reticulocyte percentage (P=7.549e-13); High light scatter reticulocyte count (P=2.948e-11); Immature reticulocyte fraction (P=5.8129e-11); Reticulocyte percentage (P=1.1537e-08) | SGPP1 |
| rs608994 | Arachidic acid 20:0 | X | SPTLC3 |
| rs3910136 | X | X | SPTLC3 |
| rs6041755 | X | X | SPTLC3 |
| rs3848754 | X | X | SPTLC3 |
| rs3848755 | X | X | SPTLC3 |
| rs13037956 | X | X | SPTLC3 |
| rs6074538 | X | X | SPTLC3 |
| rs6078866 | X | X | SPTLC3 |
| rs6074539 | X | X | SPTLC3 |
| rs6940658 | X | X | CD83 |
| rs4333409 | X | X | CD83 |
| rs2039310 | X | X | CD83 |
| rs9382948 | X | X | CD83 |
| rs6910045 | X | X | CD83 |
| rs9367828 | X | X | CD83 |
| rs9370735 | X | X | CD83 |
| rs6940973 | X | X | CD83 |
| rs1537152 | X | X | CD83 |
| rs1537151 | X | X | CD83 |
| rs9396477 | X | X | CD83 |
| rs12208698 | X | X | CD83 |
| rs12190393 | X | X | CD83 |
| rs12212956 | X | X | CD83 |
| rs12207359 | X | X | CD83 |
| rs75762794 | X | X | CD83 |
| rs2876349 | X | X | CD83 |
| rs12213267 | X | X | CD83 |
| rs115366574 | X | X | CD83 |
| rs79263173 | X | X | CD83 |
| rs4653568 | X | Mean platelet (thrombocyte) volume (P=4.7652e-12) | DEGS1 |
| rs4654000 | X | Mean platelet (thrombocyte) volume (P=4.8955e-12) | DEGS1 |
| rs9793489 | X | Mean platelet (thrombocyte) volume (P=6.0912e-12) | DEGS1 |
| rs2011117 | X | Mean platelet (thrombocyte) volume (P=6.3718e-12) | DEGS1 |
| rs908801 | X | Mean platelet (thrombocyte) volume (P=6.3548e-12) | DEGS1 |
| rs6681673 | X | Mean platelet (thrombocyte) volume (P=7.1624e-12) | DEGS1 |
| rs4654003 | X | Mean platelet (thrombocyte) volume (P=6.6526e-12) | DEGS1 |
| rs6682292 | X | Mean platelet (thrombocyte) volume (P=7.1866e-12) | DEGS1 |
| rs12038372 | X | Mean platelet (thrombocyte) volume (P=3.3948e-18); Neutrophil count (P=1.1357e-09); White blood cell (leukocyte) count (P=3.1852e-09)). | DEGS1 |
| rs6426143 | X | Mean platelet (thrombocyte) volume (P=1.9455e-16); White blood cell (leukocyte) count (P=4.4764e-10); Neutrophil count (P=1.7302e-09); Red blood cell (erythrocyte) count (P=2.3883e-09)). | DEGS1 |
| rs6682551 | X | Mean platelet (thrombocyte) volume (P=1.409e-16); White blood cell (leukocyte) count (P=2.7024e-10); Neutrophil count (P=4.8023e-09); Red blood cell (erythrocyte) count (P=1.2395e-09)). | DEGS1 |
| rs10799505 | X | Mean platelet (thrombocyte) volume (P=1.513e-16); White blood cell (leukocyte) count (P=4.7039e-10); Red blood cell (erythrocyte) count (P=5.487e-10); Neutrophil count (P=6.0446e-10)). | DEGS1 |
| rs10916403 | X | Mean platelet (thrombocyte) volume (P=1.6163e-16); White blood cell (leukocyte) count (P=4.6079e-10); Red blood cell (erythrocyte) count (P=5.9676e-10); Neutrophil count (P=6.0795e-10); Mean sphered cell volume (P=9.3161e-08). | DEGS1 |
| rs55664906 | X | Mean platelet (thrombocyte) volume (P=5.3355e-16); Red blood cell (erythrocyte) count (P=5.5676e-10); White blood cell (leukocyte) count (P=1.6824e-09); Neutrophil count (P=2.3745e-09); Mean sphered cell volume (P=6.4397e-08). | DEGS1 |
| rs12076788 | X | Mean platelet (thrombocyte) volume (P=2.1908e-16); White blood cell (leukocyte) count (P=7.174e-10); Red blood cell (erythrocyte) count (P=1.3034e-09); Neutrophil count (P=1.6361e-09). | DEGS1 |
| rs4653563 | X | Mean platelet (thrombocyte) volume (P=9.2972e-17); White blood cell (leukocyte) count (P=4.6256e-10); Neutrophil count (P=1.5481e-09); Red blood cell (erythrocyte) count (P=2.0336e-09). | DEGS1 |
| rs4653564 | X | Mean platelet (thrombocyte) volume (P=8.4965e-17); White blood cell (leukocyte) count (P=4.9323e-10); Neutrophil count (P=1.5762e-09); Red blood cell (erythrocyte) count (P=1.8868e-09). | DEGS1 |
| rs6426139 | X | Mean platelet (thrombocyte) volume (P=8.978e-17); White blood cell (leukocyte) count (P=4.5862e-10); Neutrophil count (P=1.5587e-09); Red blood cell (erythrocyte) count (P=2.0271e-09). | DEGS1 |
| rs10753454 | X | Mean platelet (thrombocyte) volume (P=1.0592e-16); White blood cell (leukocyte) count (P=4.7645e-10); Neutrophil count (P=1.5892e-09); Red blood cell (erythrocyte) count (P=1.8429e-09). | DEGS1 |
| rs7542293 | X | Mean platelet (thrombocyte) volume (P=1.0092e-16); White blood cell (leukocyte) count (P=4.5228e-10); Neutrophil count (P=1.5183e-09); Red blood cell (erythrocyte) count (P=1.8065e-09). | DEGS1 |
| rs7542474 | X | Mean platelet (thrombocyte) volume (P=9.8213e-17); White blood cell (leukocyte) count (P=4.5324e-10); Neutrophil count (P=1.5489e-09); Red blood cell (erythrocyte) count (P=1.8863e-09). | DEGS1 |
| rs6698041 | X | Mean platelet (thrombocyte) volume (P=1.0606e-16); White blood cell (leukocyte) count (P=4.7114e-10); Neutrophil count (P=1.5776e-09); Red blood cell (erythrocyte) count (P=1.8254e-09). | DEGS1 |
| rs7519433 | X | Mean platelet (thrombocyte) volume (P=9.8438e-17); White blood cell (leukocyte) count (P=3.8904e-10); Neutrophil count (P=1.2884e-09); Red blood cell (erythrocyte) count (P=2.125e-09). | DEGS1 |
| rs12730611 | X | Mean platelet (thrombocyte) volume (P=1.0549e-16); White blood cell (leukocyte) count (P=4.5558e-10); Neutrophil count (P=1.5474e-09); Red blood cell (erythrocyte) count (P=1.7702e-09). | DEGS1 |
| rs2014782 | X | Mean platelet (thrombocyte) volume (P=1.037e-16); White blood cell (leukocyte) count (P=3.6376e-10); Neutrophil count (P=7.8676e-10); Red blood cell (erythrocyte) count (P=1.4462e-09). | DEGS1 |
| rs869945 | X | Mean platelet (thrombocyte) volume (P=1.0322e-16); White blood cell (leukocyte) count (P=3.5835e-10); Neutrophil count (P=7.7447e-10); Red blood cell (erythrocyte) count (P=1.4451e-09). | DEGS1 |
| rs6691405 | X | Mean platelet (thrombocyte) volume (P=8.4246e-17); White blood cell (leukocyte) count (P=4.2562e-10); Neutrophil count (P=1.469e-09); Red blood cell (erythrocyte) count (P=2.0154e-09). | DEGS1 |
| rs6685783 | X | Mean platelet (thrombocyte) volume (P=9.2798e-17); White blood cell (leukocyte) count (P=4.0593e-10); Neutrophil count (P=1.4225e-09); Red blood cell (erythrocyte) count (P=2.0518e-09). | DEGS1 |
| rs10916355 | X | Mean platelet (thrombocyte) volume (P=8.8465e-17); White blood cell (leukocyte) count (P=3.6897e-10); Neutrophil count (P=7.8979e-10); Red blood cell (erythrocyte) count (P=1.3939e-09). | DEGS1 |
| rs66506223 | X | Mean platelet (thrombocyte) volume (P=8.4007e-17); White blood cell (leukocyte) count (P=4.1628e-10); Neutrophil count (P=1.4926e-09); Red blood cell (erythrocyte) count (P=2.1213e-09). | DEGS1 |
| rs61827699 | X | Mean platelet (thrombocyte) volume (P=8.882e-17); White blood cell (leukocyte) count (P=3.9042e-10); Neutrophil count (P=1.3987e-09); Red blood cell (erythrocyte) count (P=2.1437e-09). | DEGS1 |
| rs10916367 | X | Mean platelet (thrombocyte) volume (P=5.0835e-17); White blood cell (leukocyte) count (P=3.555e-10); Neutrophil count (P=1.21e-09); Red blood cell (erythrocyte) count (P=1.8198e-09). | DEGS1 |
| rs4653986 | X | Mean platelet (thrombocyte) volume (P=4.6912e-17); White blood cell (leukocyte) count (P=3.513e-10); Neutrophil count (P=1.1833e-09); Red blood cell (erythrocyte) count (P=1.8124e-09). | DEGS1 |
| rs7518839 | X | Mean platelet (thrombocyte) volume (P=4.9221e-17); White blood cell (leukocyte) count (P=3.4999e-10); Neutrophil count (P=1.1542e-09); Red blood cell (erythrocyte) count (P=1.8229e-09). | DEGS1 |
| rs13374070 | X | Mean platelet (thrombocyte) volume (P=4.8718e-17); White blood cell (leukocyte) count (P=2.794e-10); Neutrophil count (P=6.354e-10); Red blood cell (erythrocyte) count (P=1.0861e-09). | DEGS1 |
| rs55736782 | X | Mean platelet (thrombocyte) volume (P=4.8498e-17); White blood cell (leukocyte) count (P=2.7262e-10); Neutrophil count (P=6.1833e-10); Red blood cell (erythrocyte) count (P=1.0713e-09). | DEGS1 |
| rs997297 | X | Mean platelet (thrombocyte) volume (P=3.7928e-17); White blood cell (leukocyte) count (P=3.6642e-10); Neutrophil count (P=1.3338e-09); Red blood cell (erythrocyte) count (P=1.8344e-09). | DEGS1 |
| rs997296 | X | Mean platelet (thrombocyte) volume (P=3.1302e-17); White blood cell (leukocyte) count (P=3.2187e-10); Neutrophil count (P=1.1007e-09); Red blood cell (erythrocyte) count (P=1.5943e-09). | DEGS1 |
| rs7531891 | X | Mean platelet (thrombocyte) volume (P=6.0753e-17); White blood cell (leukocyte) count (P=2.5459e-10); Neutrophil count (P=5.6997e-10); Red blood cell (erythrocyte) count (P=9.9016e-10). | DEGS1 |
| rs1492694 | X | Mean platelet (thrombocyte) volume (P=5.3205e-17); White blood cell (leukocyte) count (P=2.5298e-10); Neutrophil count (P=5.7417e-10); Red blood cell (erythrocyte) count (P=1.0494e-09). | DEGS1 |
| rs4653991 | X | Mean platelet (thrombocyte) volume (P=5.0426e-17); White blood cell (leukocyte) count (P=3.204e-10); Neutrophil count (P=1.0394e-09); Red blood cell (erythrocyte) count (P=1.7487e-09). | DEGS1 |
| rs7546235 | X | Mean platelet (thrombocyte) volume (P=5.2812e-17); White blood cell (leukocyte) count (P=2.4989e-10); Neutrophil count (P=5.7025e-10); Red blood cell (erythrocyte) count (P=1.0533e-09). | DEGS1 |
| rs10916371 | X | Mean platelet (thrombocyte) volume (P=4.6957e-17); White blood cell (leukocyte) count (P=3.03e-10); Neutrophil count (P=9.7973e-10); Red blood cell (erythrocyte) count (P=1.7318e-09). | DEGS1 |
| rs7524705 | X | Mean platelet (thrombocyte) volume (P=4.7556e-17); White blood cell (leukocyte) count (P=2.4762e-10); Neutrophil count (P=5.7017e-10); Red blood cell (erythrocyte) count (P=1.0728e-09). | DEGS1 |
| rs1826421 | X | Mean platelet (thrombocyte) volume (P=4.5592e-17); White blood cell (leukocyte) count (P=3.3766e-10); Neutrophil count (P=1.1157e-09); Red blood cell (erythrocyte) count (P=1.733e-09). | DEGS1 |
| rs12563153 | X | Mean platelet (thrombocyte) volume (P=4.8579e-17); White blood cell (leukocyte) count (P=2.6386e-10); Neutrophil count (P=6.0969e-10); Red blood cell (erythrocyte) count (P=1.0513e-09). | DEGS1 |
| rs8328 | X | Mean platelet (thrombocyte) volume (P=5.1273e-17); White blood cell (leukocyte) count (P=3.3169e-10); Neutrophil count (P=1.1078e-09); Red blood cell (erythrocyte) count (P=1.7271e-09). | DEGS1 |
| rs7526252 | X | Mean platelet (thrombocyte) volume (P=4.3542e-17); White blood cell (leukocyte) count (P=3.5044e-10); Neutrophil count (P=1.2329e-09); Red blood cell (erythrocyte) count (P=1.7174e-09). | DEGS1 |

**Table S12. Significant results from the single SNP two-sample Mendelian randomisation analysis.**

The table depicts the significant association of CER[N(24)S(16)] as exposures to multiple blood cell count outcomes via 2SMR analysis, and that of the CER product-precursor ratio ([CER(N(24)S(19)]/CER[N(24)DS(19)]) after adjustment for 20 tests. The beta and standard error (SE) values describe the relationship between the CER traits and blood cell phenotypes.

| **Exposure** | **SNP** | **Trait** | **Outcome ID** | **beta** | **SE** | **Padj** |
| --- | --- | --- | --- | --- | --- | --- |
| CER[N(24)S(19)]/  CER[N(24)DS(19)] ratio | rs4653568 | Mean platelet volume | ukb-d-30100_irnt | 0.04 | 0.01 | 6.20E-05 |
| CER[N(24)S(16)] | rs7160525 | Mean platelet volume | ukb-d-30100_irnt | -0.08 | 0.01 | 7.48E-16 |
| CER[N(24)S(16)] | rs7160525 | Red cell distribution width | ebi-a-GCST006804 | 0.09 | 0.02 | 1.38E-08 |
| CER[N(24)S(16)] | rs7160525 | High light scatter reticulocyte  percentage of red cells | ebi-a-GCST004612 | -0.07 | 0.01 | 2.03E-05 |
| CER[N(24)S(16)] | rs7160525 | Reticulocyte fraction of red cells | ebi-a-GCST006804 | 0.09 | 0.02 | 1.38E-08 |
| CER[N(24)S(16)] | rs7160525 | Platelet count | ukb-d-30080_irnt | 0.04 | 0.01 | 8.45E-06 |
| CER[N(24)S(16)] | rs7160525 | High light scatter reticulocyte percentage | ukb-d-30290_irnt | -0.06 | 0.01 | 4.40E-10 |
| CER[N(24)S(16)] | rs7160525 | High light scatter reticulocyte count | ukb-d-30300_irnt | -0.06 | 0.01 | 4.36E-09 |
| CER[N(24)S(16)] | rs7160525 | Immature reticulocyte fraction | ukb-d-30280_irnt | -0.06 | 0.01 | 2.71E-08 |
| CER[N(24)S(16)] | rs7160525 | Reticulocyte percentage | ukb-d-30240_irnt | -0.05 | 0.01 | 1.85E-06 |

**Table S13. Plasma ceramide concentrations by carrier status in *SPTLC3*.**

The table depicts the plasma ceramide concentration (pmol/ml) by genotype carrier status of the variant rs680379 in *SPTLC3* (AA, AG, GG) in the CER species that significantly associated at GWAS with the *SPTLC3* locus*.*

| **AA** | **N(22)S(19)** | **N(23)S(20)** | **N(24)DS(19)** | **N(24)DS(20)** | **N(24)S(16)** | **N(24)S(19)** | **N(24)S(20)** | **N(25)S(20)** | **N(26)S(19)** |
| --- | --- | --- | --- | --- | --- | --- | --- | --- | --- |
| SE | 1.92 | 1.59 | 4.56 | 1.92 | 3.18 | 48.51 | 9.92 | 1.81 | 9.26 |
| Mean | 33.57 | 53.61 | 88.33 | 41.72 | 57.38 | 1512.65 | 319.23 | 40.13 | 158.61 |
| n | 148 | 148 | 148 | 148 | 148 | 148 | 148 | 148 | 148 |
|  |  |  |  |  |  |  |  |  |  |
| **AG** | **N(22)S(19)** | **N(23)S(20)** | **N(24)DS(19)** | **N(24)DS(20)** | **N(24)S(16)** | **N(24)S(19)** | **N(24)S(20)** | **N(25)S(20)** | **N(26)S(19)** |
| SE | 0.94 | 0.85 | 2.05 | 0.98 | 1.32 | 27.49 | 5.64 | 0.80 | 3.77 |
| Mean | 28.10 | 49.46 | 69.57 | 36.30 | 46.79 | 1294.50 | 291.75 | 33.07 | 116.50 |
| n | 442 | 442 | 442 | 442 | 442 | 442 | 442 | 442 | 442 |
|  |  |  |  |  |  |  |  |  |  |
| **GG** | **N(22)S(19)** | **N(23)S(20)** | **N(24)DS(19)** | **N(24)DS(20)** | **N(24)S(16)** | **N(24)S(19)** | **N(24)S(20)** | **N(25)S(20)** | **N(26)S(19)** |
| SE | 0.64 | 0.68 | 1.40 | 0.72 | 1.18 | 24.38 | 4.60 | 0.67 | 2.79 |
| Mean | 20.41 | 43.58 | 51.46 | 29.76 | 38.92 | 1028.83 | 244.97 | 29.13 | 92.50 |
| n | 409 | 409 | 409 | 409 | 409 | 409 | 409 | 409 | 409 |

**Table S14. Plasma *N*-acyl ethanolamine concentrations by carrier status in *FAAH*.**

The table depicts the plasma *N*-acyl ethanolamine concentration (pg/ml) by genotype carrier status of the variant rs324420 in *FAAH* (AA, AC, CC) in the NAE species that significantly associated at GWAS with the *FAAH* locus.

| **AA** | **PEA** | **VEA** | **DHEA** | **LEA** |
| --- | --- | --- | --- | --- |
| SE | 219.39 | 50.57 | 72.07 | 88.13 |
| mean | 2193.62 | 358.10 | 519.08 | 792.31 |
| n | 51 | 51 | 51 | 51 |
|  |  |  |  |  |
| **AC** | **PEA** | **VEA** | **DHEA** | **LEA** |
| SE | 81.77 | 17.16 | 17.04 | 30.99 |
| mean | 2015.33 | 286.22 | 379.23 | 661.01 |
| n | 310 | 310 | 310 | 310 |
|  |  |  |  |  |
| **CC** | **PEA** | **VEA** | **DHEA** | **LEA** |
| SE | 50.96 | 8.70 | 9.99 | 18.92 |
| mean | 1792.92 | 227.21 | 321.07 | 583.94 |
| n | 638 | 638 | 638 | 638 |

**Supplemental Figures**

**A**

**B**

**Figure S1.** Intra-correlation analysis of ceramides, sphingoid bases, and related sphingolipids, and *N*-acyl ethanolamines.

The figure depicts the assessment of the relatedness within the two classes of lipids (A: CER, B: NAE) for the 999 plasma samples studied. The tool rquery.cormat was used in R, which takes into account strength of relationship (correlation coefficient; depicted as a scale of colours) and P-value (size of circle produced). The correlation was completed on the covariate-adjusted, standardised residuals used for genetic analyses.

**Figure S2.** The frequency of individuals in each collected family that were analysed in this lipidomics study.

The graph depicts the range of individuals (1-24) with plasma available for lipidomics analyses. 999 individuals were assessed from 196 families. The mean number of individuals from each family assessed for mediator lipidomics was 5.


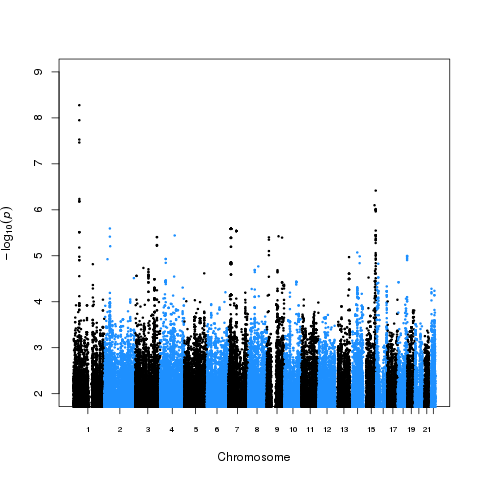


**Figure S3.** Manhattan plot of an example NAE species (PEA) associated at GWAS with SNPs in *FAAH* on chromosome 1.

The figure shows a Manhattan plot of GWAS results for PEA lipid species highlighting significant SNPs at chromosome 1 at the fatty acid amide hydrolase gene (*FAAH*) in 993 plasma samples.


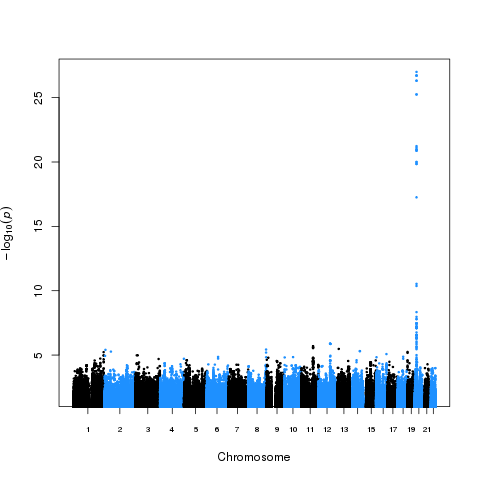


**Figure S4.** Manhattan plot of an example CER species N(24)S(19) associated at GWAS with SNPs in *SPTLC3* on chromosome 20.

The figure shows a Manhattan plot of GWAS results for CER[N(24)S(19)], highlighting significant SNPs at chromosome 20 at the serine palmitoyltransferase gene (*SPTLC3*) in 991 plasma samples. ****

**Figure S5.** LocusZoom plot depicting the GWAS association of CER[N(26)S(19)] to the locus at CD83 on chromosome 6.

The figure depicts the GWAS association of this CER species with the locus of the leukocyte surface marker, CD83 in 991 plasma samples. The r^2^ for each SNP is depicted in colour. This finding was supported by three significant genotyped SNPs. The plot was created using the LocusZoom plot tools at http://locuszoom.sph.umich.edu/.

**Figure S6.** LocusZoom plot depicting the GWAS association of the ratio of CER[N(24)S(19)] to its precursor CER[N(24)DS(19)], at the locus of *DEGS1* on chromosome 1.

The figure depicts the GWAS association of this CER species with the locus of the desaturase enzyme, DEGS1 in 991 plasma samples. The r^2^ for each SNP is depicted in colour. This finding was supported by two significant genotyped SNPs. The plot was created using the LocusZoom plot tools at http://locuszoom.sph.umich.edu/.

**Figure S7.** LocusZoom plot depicting the GWAS association of CER[N(24)S(16)] to the locus at *SGPP1* on chromosome 14.

The figure depicts the GWAS association of this CER species with the locus of the sphingosine 1-phosphate phosphatase enzyme, SGPP1 in 992 plasma samples. The r^2^ for each SNP is depicted in colour. This finding was supported by rs7157785, a GWAS significant genotyped SNP. The plot was created using the LocusZoom plot tools at http://locuszoom.sph.umich.edu/.
